# Early Secretory Pathway-Associated Proteins SsEmp24 and SsErv25 Are Involved in Morphogenesis and Pathogenicity in a Filamentous Phytopathogenic Fungus

**DOI:** 10.1128/mBio.03173-21

**Published:** 2021-12-21

**Authors:** Chong Xie, Qingna Shang, Chenmi Mo, Yannong Xiao, Gaofeng Wang, Jiatao Xie, Daohong Jiang, Xueqiong Xiao

**Affiliations:** a State Key Laboratory of Agricultural Microbiology, Huazhong Agricultural University, Wuhan, Hubei Province, China; b Hubei Key Laboratory of Plant Pathology, College of Plant Science and Technology, Huazhong Agricultural University, Wuhan, Hubei Province, China; c Hubei Hongshan Laboratory, Wuhan, Hubei Province, China; Cornell University

**Keywords:** early secretory pathway, *Sclerotinia sclerotiorum*, infection cushion, p24 protein family, pathogenesis

## Abstract

Proper protein secretion is critical for fungal development and pathogenesis. However, the potential roles of proteins involved in the early secretory pathway are largely undescribed in filamentous fungi. p24 proteins are cargo receptors that cycle between the endoplasmic reticulum (ER) and Golgi apparatus in the early secretory pathway and recruit cargo proteins to nascent vesicles. This study characterized the function of two p24 family proteins (SsEmp24 and SsErv25) in a phytopathogenic fungus, Sclerotinia sclerotiorum. Both *SsEmp24* and *SsErv25* were upregulated during the early stages of *S. sclerotiorum* infection. Δ*SsEmp24* mutant and Δ*SsErv25* mutant displayed abnormal vegetative growth and sclerotium formation, were defective in infection cushion formation, and showed lower virulence on host plants. Δ*SsEmp24* mutant had a more severe abnormal phenotype than Δ*SsErv25* mutant, implying that SsEmp24 could play a central role in the early secretory pathway. Similar to their *Saccharomyces cerevisiae* counterparts, SsEmp24 interacted with SsErv25 and predominantly colocalized in the ER or nuclear envelope. The absence of SsEmp24 or SsErv25 led to defective in protein secretion in *S. sclerotiorum*, including the pathogenicity-related extracellular hydrolytic enzymes and effectors. It is proposed that SsEmp24 and SsErv25, components in the early secretory pathway, are involved in modulating morphogenesis and pathogenicity in *S. sclerotiorum* by mediating protein secretion.

## INTRODUCTION

Sclerotinia sclerotiorum is a destructive phytopathogenic fungus with worldwide distribution (1). It causes sclerotinia disease by infecting a wide range of hosts, including rapeseed, soybean, sunflower, and horticultural plants (2, 3). At the early stage of infection, *S*. *sclerotiorum* hyphae form the infection structures termed “compound appressoria” or “infection cushions” to penetrate host tissues (4, 5). The infection structure is predicted to continuously secret certain proteins, including effectors and extracellular hydrolytic enzymes, to promote pathogenic fungal invasion and proliferation (6, 7). Protein secretion in eukaryotes depends mainly on the conventional endoplasmic reticulum (ER)-to-Golgi secretory pathway, where proteins are first translocated into the ER, then transported by ER-derived coated vesicles to the Golgi apparatus for further processing, and ultimately sent to their destination (8–10). The ER-derived coated vesicles consist of COPII and COPI, which are responsible for anterograde and retrograde transport between the ER and the Golgi apparatus, respectively (11, 12). Cargo receptors located on the membrane of the coated vesicles are responsible for recruiting cargo proteins to nascent vesicles (11, 13). For example, the cargo receptor Ssp120 in Saccharomyces cerevisiae is packaged into COPII to participate in the early secretory pathway (14, 15). Our previous study found that the Ssp120 homologous protein in *S. sclerotiorum*, Ss-Caf1 (compound appressorium formation-related protein 1), was involved in pathogenicity (5). This finding implies that the cargo receptor could be a critical pathogenic-related factor and that the early secretory pathway may be involved in regulating pathogenicity.

p24 proteins were initially identified as constituents of the early secretory compartment membranes and are abundant membrane proteins on COPI and COPII vesicles (11, 16, 17). They are widely present in fungi, plants, animals, and human and are divided into four subfamilies: p24α, p24β, p24γ, and p24δ (18, 19). In eukaryotic cells, the p24 family is proposed to function as a cargo receptor and is required for efficient protein sorting (16, 20). Members of the p24 family are proteins of approximately 24 kDa and share a similar overall membrane topology, including a N-terminal lumenal domain, a single-pass transmembrane region, and a short C-terminal cytoplasmic tail sequence (21). p24 protein deficiencies decrease the ability to retain proteins and allow the secretion and transport of misfolded proteins (11, 22, 23). Emp24 protein (Emp24p) is the first reported member of the p24 family that functions in actively sorting cargos in S. cerevisiae through binding with Erv25p (24, 25). The absence of p24β (Emp24p) or p24δ (Erv25p) results in delaying transport of invertase and the glycosylphosphatidylinositol-anchored protein (GPI-AP) Gas1p in S. cerevisiae (24, 25). Similarly, p24 proteins are involved in ER export and transport of GPI-APs to the plasma membrane in Arabidopsis thaliana (26). Furthermore, they play specific roles in mammalian health, including embryonic development, insulin secretion, Alzheimer's disease, and nonalcoholic fatty liver disease (27–30). In a recent study, the early secretory pathway associates with assemblage and excretion of infective particles of severe acute respiratory syndrome coronavirus 2 (31). However, the identity of putative cargos of cargo receptors in phytopathogenic fungi is still elusive, and the function of the p24 proteins in filamentous phytopathogenic fungal pathogenicity remains to be elucidated.

In this study, we characterized that the biological functions of two p24 proteins, SsEmp24 and SsErv25, were associated with morphogenesis and pathogenicity in *S*. *sclerotiorum* through the early secretory pathway. Moreover, the absence of SsEmp24 or SsErv25 resulted in the declining ability of protein secretion. Thus, our study provided further information on the biological functions of the early secretory pathway in phytopathogenic fungi and a better understanding of the pathogenesis mechanism of fungal pathogens.

## RESULTS

### Characterization of p24 proteins in *S*. *sclerotiorum* and other filamentous fungi.

**FIG 1:**
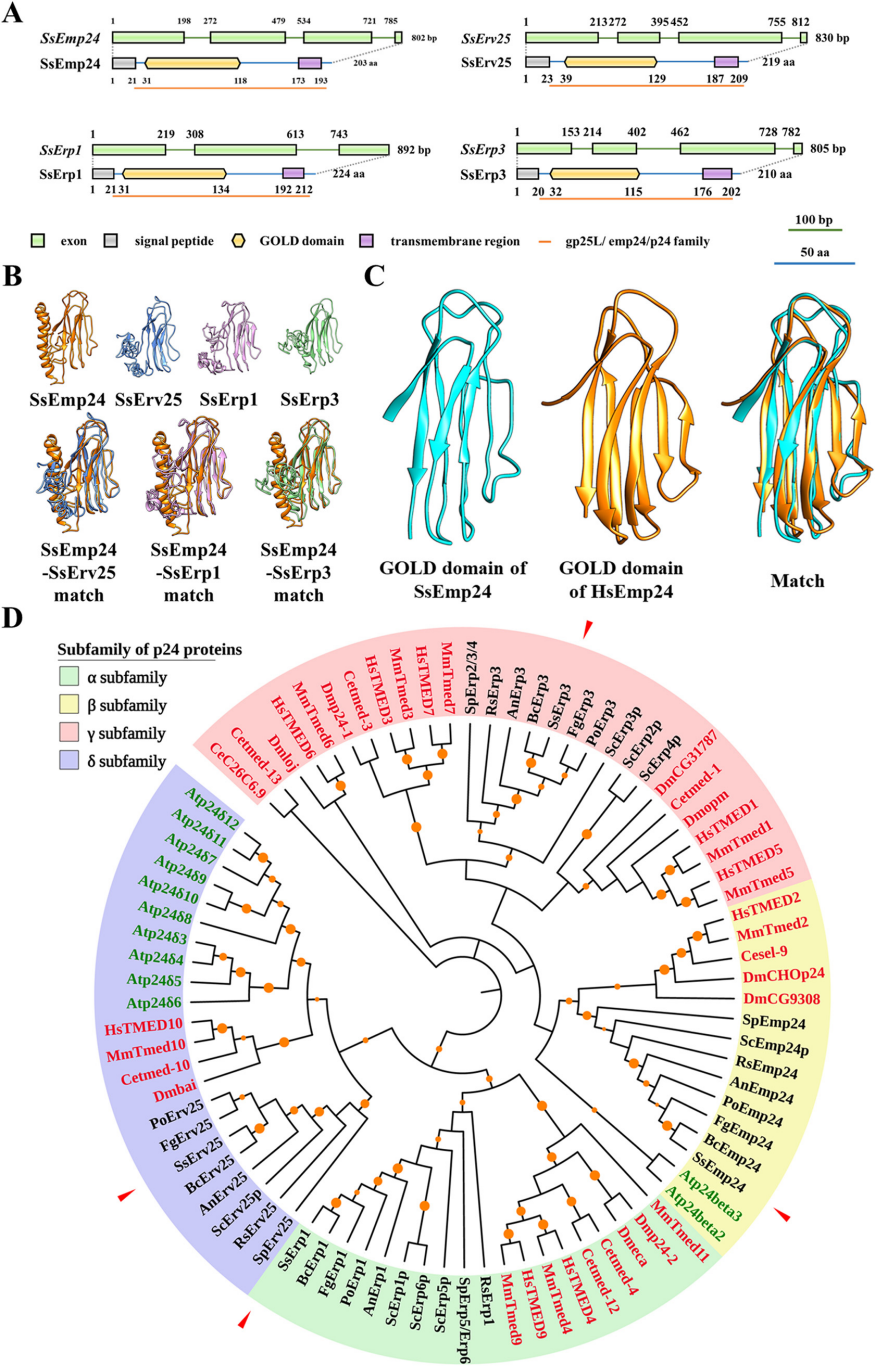
Characterizations of p24 proteins in *S. sclerotiorum*. (A) Conserved domains of p24 proteins in *S. sclerotiorum* are shown in proportion to the length of the nucleotide and amino acid sequences. (B) Three-dimensional structures of p24 proteins in *S. sclerotiorum*. The amino acid sequences of p24 proteins were used to predict the three-dimensional structures (upper panel). For structure match, the protein structures of SsErv25, SsErp1, and SsErp3 were matched and aligned with that of SsEmp24 (lower panel). (C) The three-dimensional structure model of the conserved GOLD domain in SsEmp24 was matched with the crystal structure of the p24beta1 GOLD domain (PDB ID 5AZW). (D) Phylogenetic analysis of p24 proteins in typical species. The p24 proteins of animals, plants, and fungi are represented in red, green, and black type, respectively. Different colors in the background indicate distinct subfamilies of p24 proteins, and p24 proteins from *S. sclerotiorum* are indicated by red arrows. Information including abbreviations and accession numbers of individual proteins are listed in Table S1 in the supplemental material. Hs, Homo sapiens; Mm, Mus musculus; Dm, Drosophila melanogaster; Ce, Caenorhabditis elegans; At, Arabidopsis thaliana; Sc, Saccharomyces cerevisiae; Sp, Schizosaccharomyces pombe; Rs, Rhizoctonia solani; An, Aspergillus nidulans; Po, *Pyricularia oryzae* (Magnaporthe oryzae); Fg, Fusarium graminearum; Bc, *Botrytis cinerea*; Ss, Sclerotinia sclerotiorum.

Four p24 proteins (SsErp1, SsEmp24, SsErp3, and SsErv25) were identified from *S. sclerotiorum* with reference to the known p24 protein sequences from S. cerevisiae. The coding genes of homologous *S*. *sclerotiorum* p24 proteins contain three to four exons and vary from 802 to 892 bp in length, and the sizes of their encoded proteins range from 203 to 224 amino acids (Fig. 1A). All p24 proteins in *S*. *sclerotiorum* contain a signal peptide in the N terminus, a lumenal domain of approximately 160 amino acids, a transmembrane region, and a 10- to 12-amino-acid cytosolic tail in the C terminus (see Fig. S1A in the supplemental material). The predicted three-dimensional structures of all *S*. *sclerotiorum* p24 proteins harbor a typical and highly conserved GOLD domain consisting of a β-sandwich fold (Fig. 1B), and the GOLD domain of SsEmp24 is well matched with the corresponding crystal structure of human p24beta1 protein GOLD domain (PDB ID 5AZW) (Fig. 1C) (32). Intriguingly, the alignment shows that loop 1 of the GOLD domain in filamentous fungi is conserved and yet differs from that in yeast species (Fig. S1B).

10.1128/mBio.03173-21.1 FIG S1 (A) Multiple alignment of p24 proteins identified from S. cerevisiae, C. albicans, and *S. sclerotiorum*. Signal peptide, transmembrane region, and tail of p24 protein are labeled above or below the sequences. α and β mean the secondary structures α helix and β fold, respectively. Different colors shading residues represent the different percentages of conserved sequence: orange, 100%; cyan, 80%; and yellow, 60%. Sc, Saccharomyces cerevisiae; Ca, Candida albicans; Ss, Sclerotinia sclerotiorum. (B) Multiple alignment of GOLD domain in Emp24p from yeast species and filamentous fungi. Loop 1 and loop 2 of the GOLD domain are labeled above the sequences. Different colors shading residues represent the different percentages of conserved sequence: orange, 100%; cyan, 80%; and yellow, 60%. Sc, Saccharomyces cerevisiae; Sb, Saccharomyces boulardii; Ca, Candida albicans; Po, *Pyricularia oryzae *(Magnaporthe oryzae); Fg, Fusarium graminearum; Bc, *Botrytis cinerea*; Ss, Sclerotinia sclerotiorum. Download FIG S1, TIF file, 2.3 MB. Copyright © 2021 Xie et al. 2021 Xie et al. https://creativecommons.org/licenses/by/4.0/ This content is distributed under the terms of the Creative Commons Attribution 4.0 International license.

10.1128/mBio.03173-21.7 TABLE S1 Information including accession numbers and abbreviations of genes and proteins in this study. Download Table S1, XLSX file, 0.01 MB. Copyright © 2021 Xie et al. 2021 Xie et al. https://creativecommons.org/licenses/by/4.0/ This content is distributed under the terms of the Creative Commons Attribution 4.0 International license.

Phylogenetic analysis revealed that p24 proteins are widely present in vastly different species, including animals, plants, and fungi, and are divided into four subfamilies: p24α, p24β, p24γ, and p24δ (Fig. 1D). SsErp1, SsEmp24, SsErp3, and SsErv25 from *S. sclerotiorum* belong to the α, β, γ, and δ subfamilies, respectively. Moreover, the homologous p24 proteins from filamentous fungi, including Rhizoctonia solani, Aspergillus nidulans, Magnaporthe oryzae, Fusarium graminearum, Botrytis cinerea, and *S. sclerotiorum*, which had the same p24 protein number and subfamily distribution, were in the same cluster. In contrast, the p24 proteins from yeast species belonged to another cluster. This implies that the p24 proteins in filamentous and nonfilamentous fungi might play different functions.

### *SsEmp24* and *SsErv25* had higher expression levels at the initial stage of infection cushion formation than during vegetative growth.

**FIG 2:**
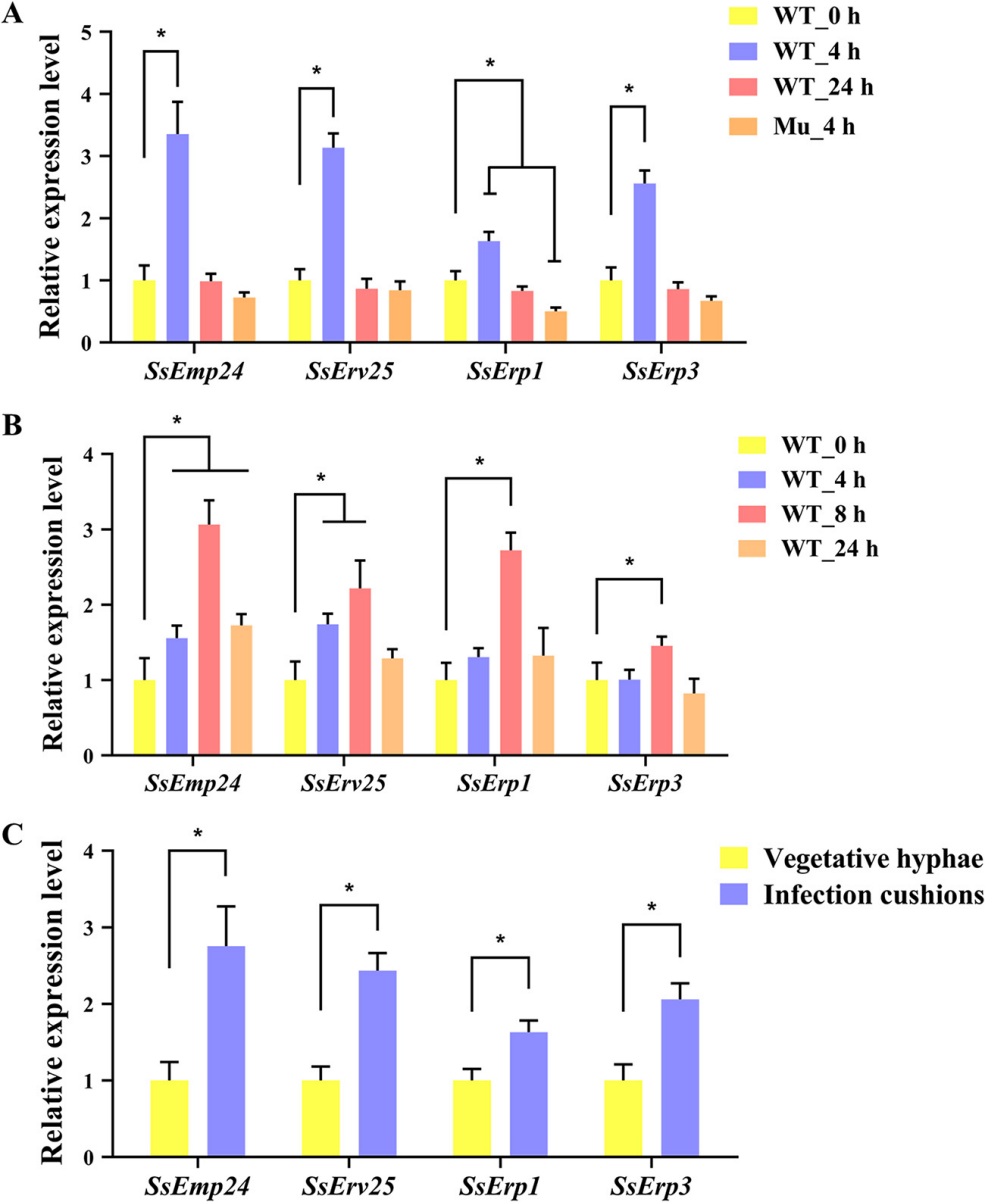
Expression levels of p24 genes in the wild-type strain (WT) and the *Ss-caf1* gene disruption mutant (Mu) in *S. sclerotiorum*. (A) The wild-type strain (WT) and the *Ss-caf1* gene disruption mutant (Mu) were inoculated on Parafilm. (B) The wild-type strain (WT) was inoculated on intact rapeseed leaves. Time points post inoculation include 4 h, 8 h, and 24 h. The wild-type strain inoculated for 0 h was used as a control. (C) Infection cushions formed on Parafilm by the wild-type strain were collected as samples, and vegetative hyphae formed on cellophane were used as a control. The *actin* gene was used as the reference gene to standardize data. Data were analyzed by two-way ANOVA, and error bars represent the SD. *, *P < *0.01 in the variance analysis (*n* = 4, three independent experiments).

Our previous study demonstrated that the pathogenicity-associated gene *Ss-caf1*, whose homolog in S. cerevisiae participated in the early secretory pathway (14, 15), was upregulated at the initial stage of infection cushion formation (5). Similar to *Ss-caf1*, four p24 genes in wild-type *S. sclerotiorum*, especially *SsEmp24* and *SsErv25*, were upregulated at the initial stage of infection cushion formation whether on Parafilm or host plant (Fig. 2A and B). In addition, the expression level of p24 genes in the *Ss-caf1* disruption mutant was assessed by quantitative reverse transcription-PCR (RT-qPCR) (Fig. 2A). Compared with the wild-type strain, the p24 genes in the *Ss-caf1* disruption mutant at the initial stage of infection cushion formation were significantly downregulated (Fig. 2A). Moreover, the expression of p24 genes was significantly higher in infection cushion structures than in vegetative hyphae (Fig. 2C). These results suggested that p24 proteins are likely involved in the infection cushion formation in *S. sclerotiorum*.

### Deletion of *SsEmp24* or *SsErv25* impairs vegetative growth and sclerotial development.

To determine the biological function of SsEmp24 and SsErv25 in *S. sclerotiorum*, knockout (KO) mutants and complementary (COM) transformants of *SsEmp24* and *SsErv25* were obtained and analyzed. The KO mutants were verified by PCR (Fig. S2A and B) and Southern blotting (Fig. S2C and D). *SsEmp24* KO (Δ*SsEmp24*) mutants were termed 24KO-1, 24KO-2, and 24KO-3, and *SsErv25* KO (Δ*SsErv25*) mutants were termed 25KO-1 and 25KO-2. The genetic complementation transformants for *SsEmp24* and *SsErv25* were identified using PCR and RT-PCR, and the COM transformants were termed 24Com and 25Com (Fig. S2E and F).

10.1128/mBio.03173-21.2 FIG S2 Verification of Δ*SsEmp24* mutants, Δ*SsErv25* mutants, and complementary transformants. (A and B) PCR verification of Δ*SsEmp24* mutants (A) and Δ*SsErv25* mutants (B). (C and D) Southern blot verification of Δ*SsEmp24* mutants and Δ*SsErv25* mutants. Probe 1 is a 3′ flanking sequence of the *SsEmp24* gene, probe 2 is a fragment of the *hph* gene, and a fragment of the *SsErv25* gene is used as probe 3. (E and F) PCR verification of the *SsEmp24* (E) and *SsErv25* (F) complementary transformants. Download FIG S2, TIF file, 1.2 MB. Copyright © 2021 Xie et al. 2021 Xie et al. https://creativecommons.org/licenses/by/4.0/ This content is distributed under the terms of the Creative Commons Attribution 4.0 International license.

**FIG 3:**
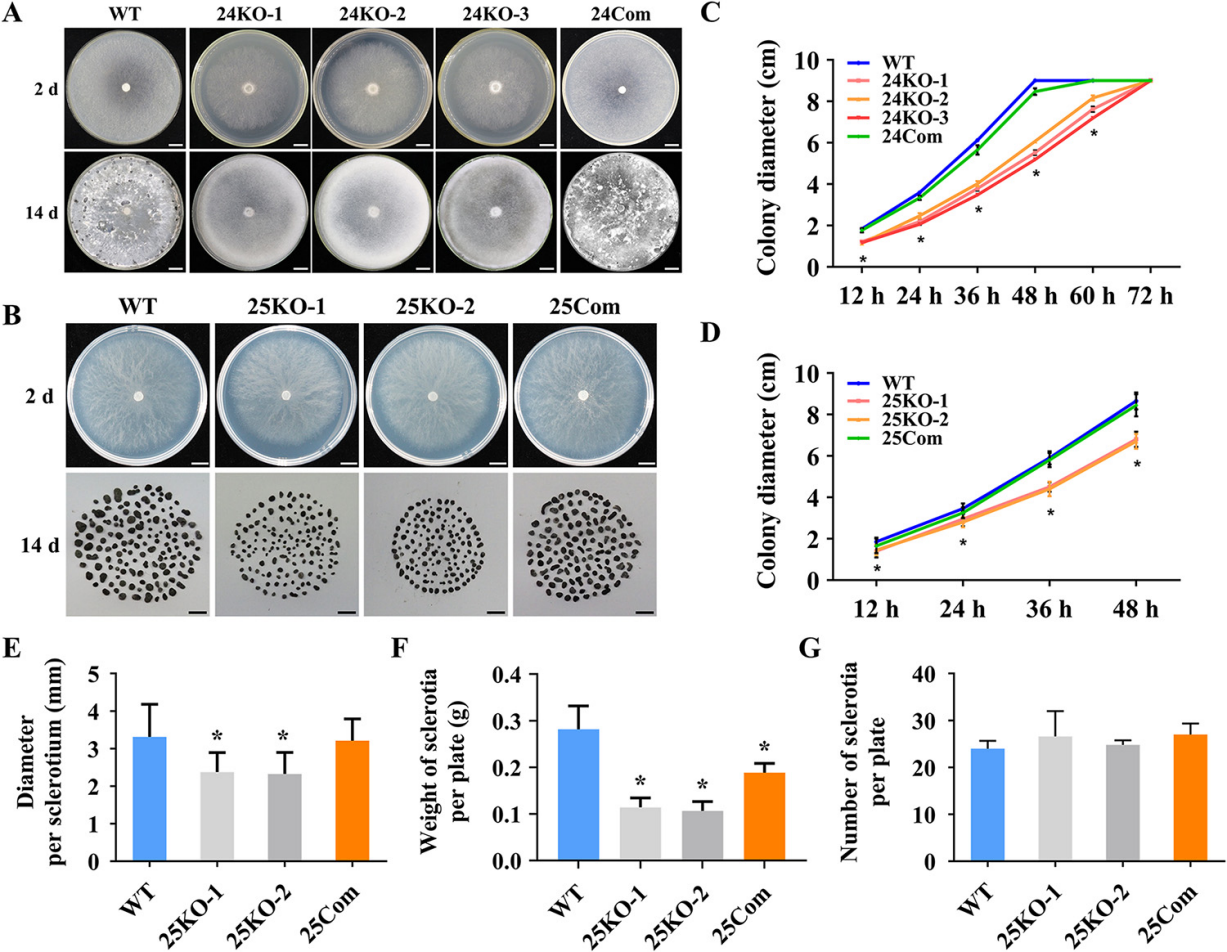
Colony morphologies of Δ*SsEmp24* mutants or Δ*SsErv25* mutants and complementary transformants on the PDA plate. (A) Colony morphologies of Δ*SsEmp24* mutants (24KO-1, 24KO-2, and 24KO-3), complementary transformant (24Com), and wild-type strain (WT) at 20°C for 2 days or 14 days. Scale bar, 1 cm. (B) Colony morphologies of Δ*SsErv25* mutants (25KO-1 and 25KO-2), complementary transformant (25Com), and wild-type strain (WT) were observed at 20°C for 2 days. The mature sclerotia collected from five PDA plates of each isolate cultured at 20°C for 14 days are displayed on the bottom row. Scale bar, 1 cm. (C and D) The colony diameters of Δ*SsEmp24* mutants, Δ*SsErv25* mutants, and complementary transformants were evaluated every 12 h until 3 dpi. Data were analyzed by two-way ANOVA (*n* = 3, three independent experiments). (E to G) The diameter per sclerotium (E), the sclerotia weight per plate (F), and the sclerotia number per plate of the sclerotia collected from the PDA plates of Δ*SsErv25* mutants and complementary transformant. Data were analyzed by one-way ANOVA, and error bars indicate the SD (*n* = 3, three independent experiments). *, *P* < 0.01 in the variance analysis.

To evaluate the role of *SsEmp24* and *SsErv25* in *S. sclerotiorum* development, colony morphology and sclerotium formation were observed. The vegetative growth of the Δ*SsEmp24* mutants or Δ*SsErv25* mutants were remarkably inhibited compared with the wild-type strain and complementary transformants (Fig. 3). Δ*SsEmp24* mutants were incapable of forming sclerotia at 14 days post inoculation (dpi), while the wild type formed normal sclerotia (Fig. 3A and B). The number of mature sclerotia was not dramatically different between Δ*SsErv25* mutants and the wild type (Fig. 3G). Nevertheless, the diameter per sclerotium and the weight of sclerotia per plate of Δ*SsErv25* mutants were significantly smaller and less than those of the wild type (Fig. 3E and F). In addition, complementary transformants of *SsEmp24* and *SsErv25* restored the wild-type phenotypes of growth and sclerotia development (Fig. 3).

### SsEmp24 and SsErv25 are associated with pathogenicity.

**FIG 4:**
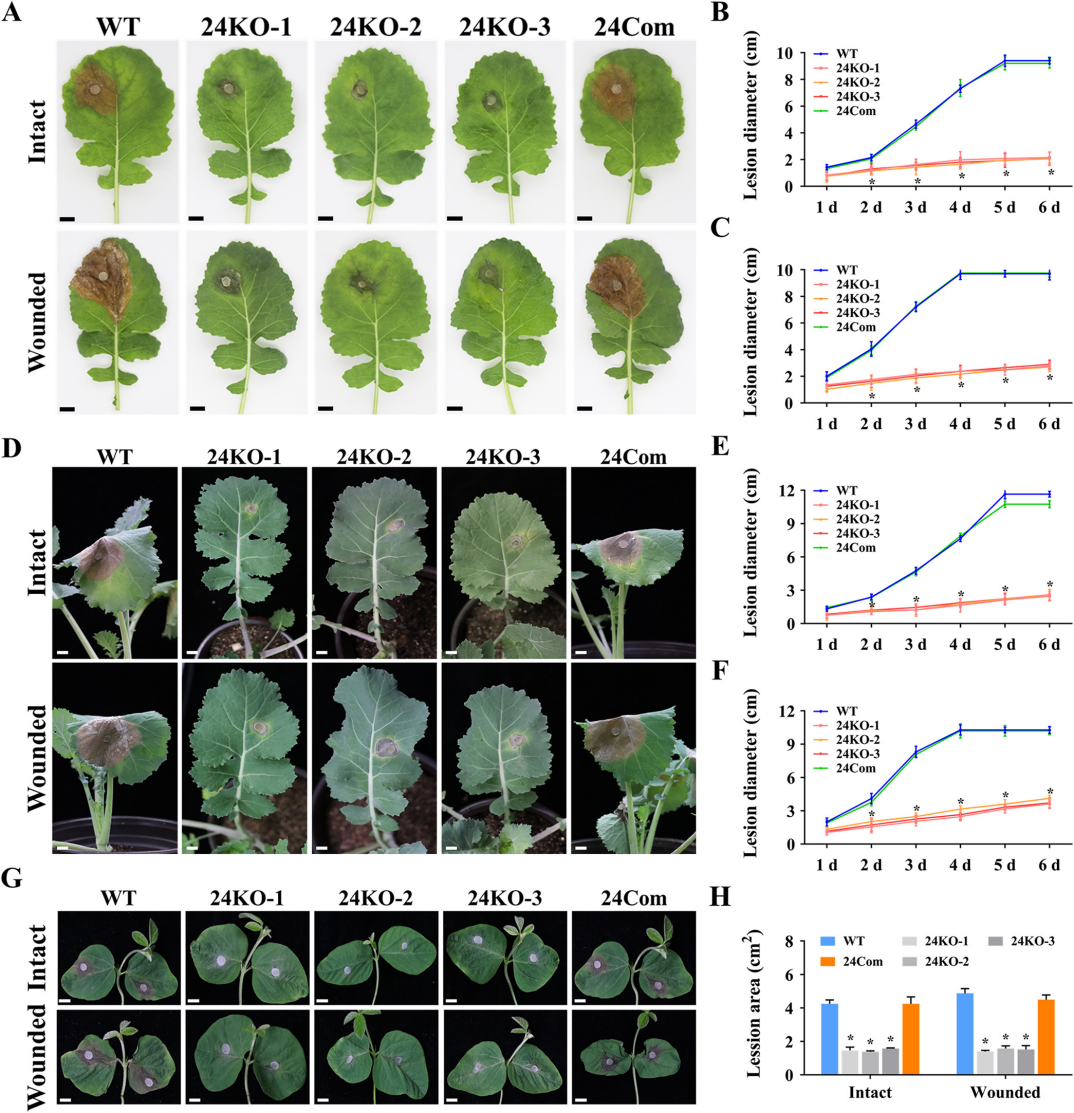
Virulence assay of Δ*SsEmp24* mutants and complementary transformant on host plants. (A) The virulence of the wild type (WT), Δ*SsEmp24* mutants (24KO-1, 24KO-2, and 24KO-3), and complementary transformant (24Com) was assayed on the intact or wounded detached rapeseed leaves. Strains were inoculated on rapeseed leaves for 2 days at 20°C. Scale bar, 1 cm. (B and C) Lesion diameters were measured on intact or wounded detached rapeseed leaves daily until 6 dpi. (D) Virulence of the individual strains for intact or wounded living rapeseed plants at 2 dpi. Scale bar, 1 cm. (E and F) Lesion diameters were measured on intact or wounded living rapeseed plants daily until 6 dpi. (G) Virulence of strains for intact or wounded living soybean plants at 2 dpi. Scale bar, 1 cm. (H) The lesion areas on intact or wounded living soybean plants at 2 dpi were counted by the software ImageJ 1.52a. Data were analyzed by two-way ANOVA, and error bars represent the SD. *, *P < *0.01 in the variance analysis (*n* = 4, four independent experiments).

**FIG 5:**
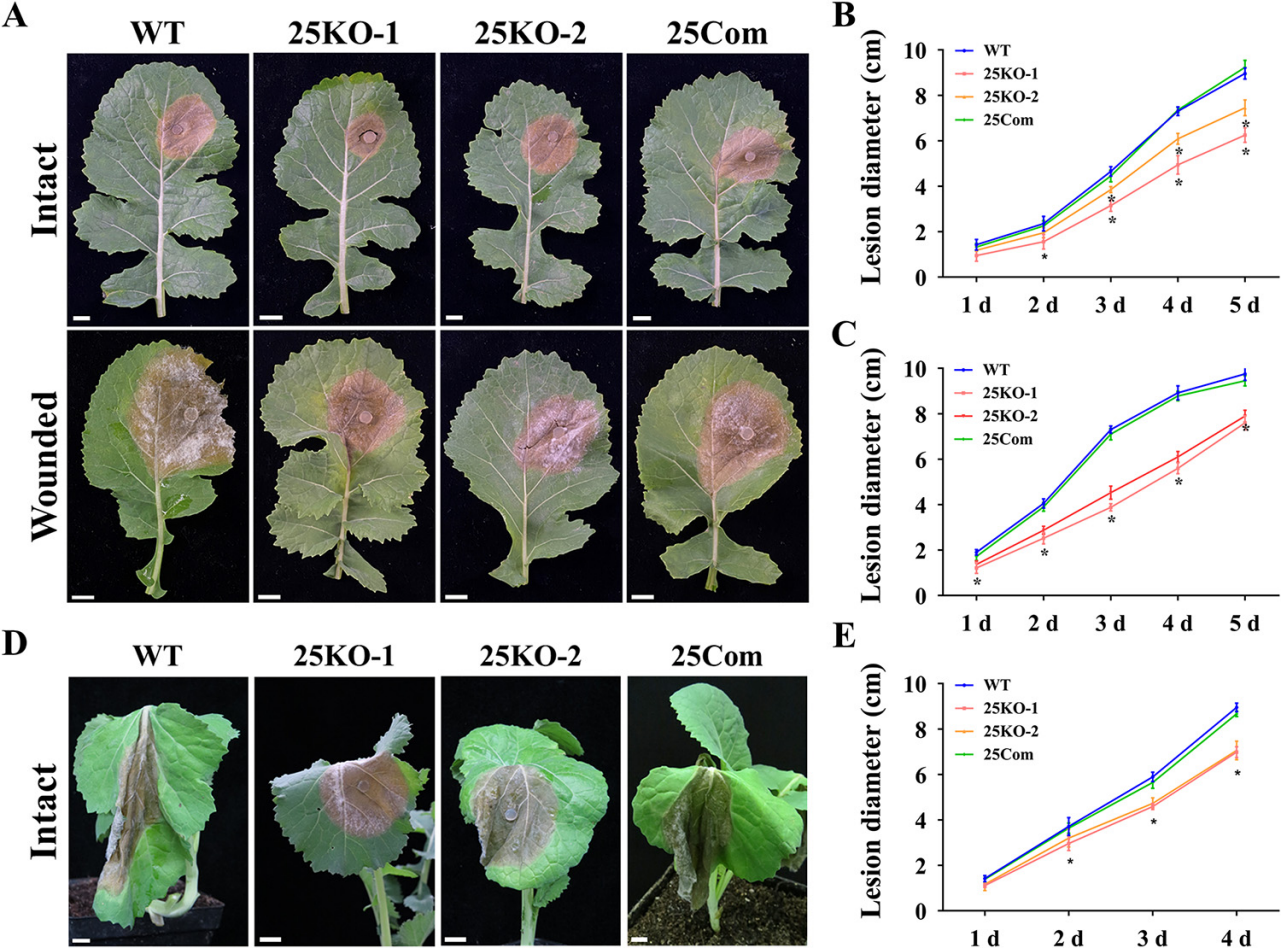
Virulence assay of Δ*SsErv25* mutants on host plants. (A) The virulence of the wild type (WT), Δ*SsErv25* mutants (25KO-1 and 25KO-2), and complementary transformant (25Com) was assayed on the intact or wounded detached rapeseed leaves. Strains were inoculated on rapeseed leaves for 2 days at 20°C. Scale bar, 1 cm. (B and C) Lesion diameters were measured on intact or wounded detached rapeseed leaves daily until 5 dpi. (D) Virulence of strains on intact living rapeseed plants at 3 dpi. Scale bar, 1 cm. (E) Lesion diameters were measured on intact living rapeseed plants daily until 4 dpi. Data were analyzed by two-way ANOVA, and error bars represent the SD. *, *P < *0.01 in the variance analysis (*n* = 4, four independent experiments).

To analyze the role of *SsEmp24* and *SsErv25* in pathogenicity, the individual strains of *S. sclerotiorum* were inoculated on detached leaves or living host plants, rapeseed or soybean. Both Δ*SsEmp24* mutants and Δ*SsErv25* mutants had dramatically reduced pathogenicity on intact and wounded host leaves compared with that of the wild type and complementary transformants (Fig. 4 and 5). In addition, the lesions caused by Δ*SsEmp24* mutants were smaller than that of Δ*SsErv25* mutants and failed to further develop after 3 dpi (Fig. 4 and 5). Surprisingly, the lesion caused by Δ*SsEmp24* mutants on wounded leaves showed no significant difference with that on intact leaves (Fig. 4; Fig. S3). In contrast, the wild type caused a significantly larger lesion on wounded leaves than on intact leaves (Fig. 4; Fig. S3). This indicated that *SsEmp24* deletion caused a significant impact on the proliferation stage of the pathogenicity process.

10.1128/mBio.03173-21.3 FIG S3 Virulence analysis of Δ*SsEmp24* mutants on wounded detached rapeseed leaves. The wild type (WT), Δ*SsEmp24* mutants (24KO-1, 24KO-2, and 24KO-3), and complementary transformant (24Com) were inoculated on rapeseed leaves for 2 days at 20°C. The lesion diameters on wounded leaves were measured at 2 dpi and are compared with those on intact leaves. Data were analyzed by two-way ANOVA, and error bars represent the SD. *, *P < *0.01 in the variance analysis (*n* = 4, four independent experiments). Download FIG S3, TIF file, 0.6 MB. Copyright © 2021 Xie et al. 2021 Xie et al. https://creativecommons.org/licenses/by/4.0/ This content is distributed under the terms of the Creative Commons Attribution 4.0 International license.

### SsEmp24 and SsErv25 are related to infection cushion formation and acid accumulation.

**FIG 6:**
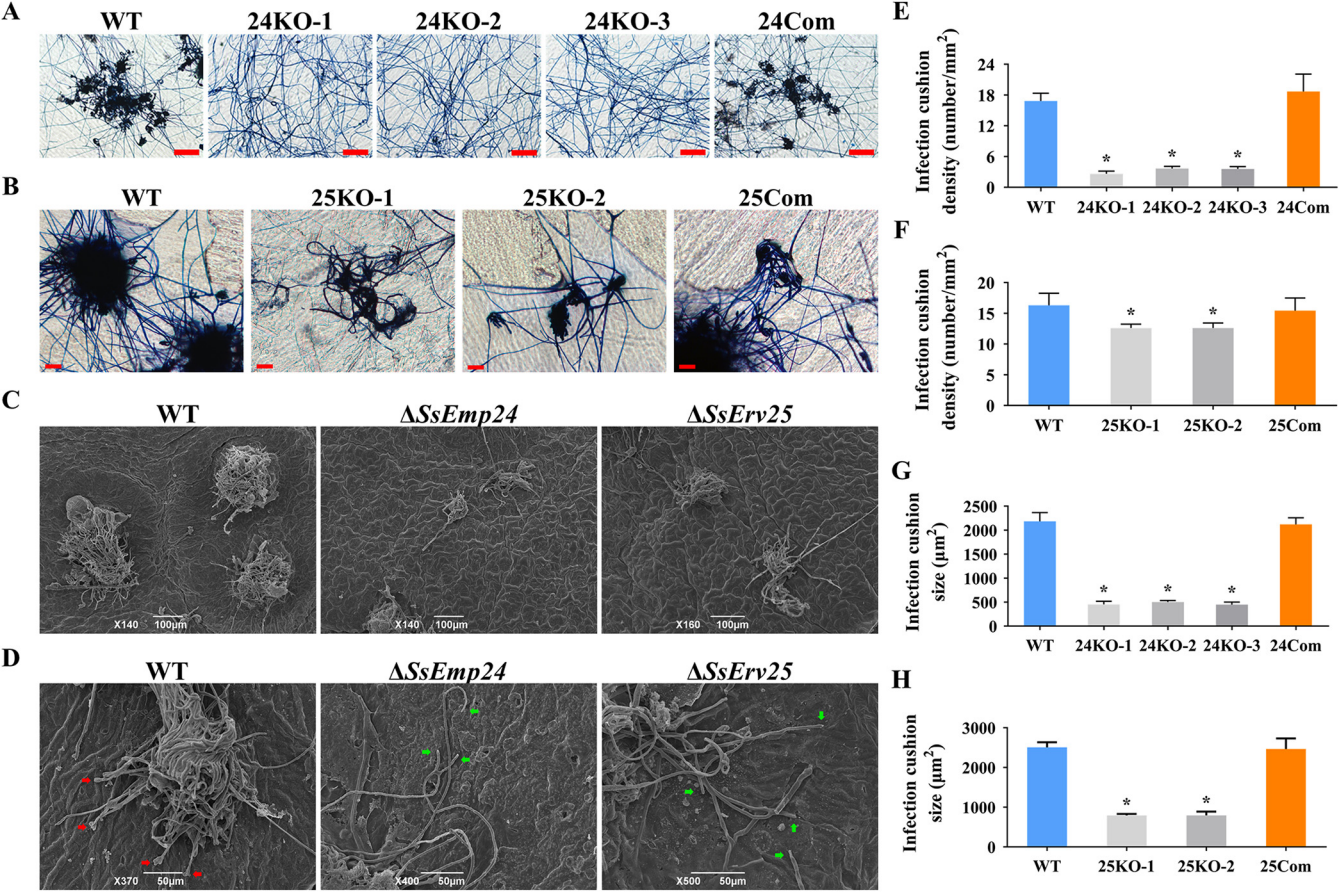
Infection cushion formation assays. (A) Infection cushions formed by the wild type (WT), Δ*SsEmp24* mutants (24KO-1, 24KO-2, and 24KO-3), and complementary transformant (24Com) on Parafilm were observed by microscopy. Scale bar, 100 μm. (B) Infection cushions formed by the wild type (WT), Δ*SsErv25* mutants (25KO-1 and 25KO-2), and complementary transformant (25Com) on Parafilm were observed by microscopy. Scale bar, 20 μm. (C) Infection cushions formed by the wild type (WT), Δ*SsEmp24* mutant, and Δ*SsErv25* mutant on intact rapeseed leaves were observed by scanning electron microscopy (SEM). (D) The tips of infection cushions formed by the wild type (WT), Δ*SsEmp24* mutant, and Δ*SsErv25* mutant on intact rapeseed leaves were observed by SEM. Red arrows indicate the new tips of infection cushions formed by the wild type, and green arrows indicate the hyphal tips of infection cushions formed by Δ*SsEmp24* mutant and Δ*SsErv25* mutant. (E and F) Density of infection cushion formed by Δ*SsEmp24* mutants (E) and Δ*SsErv25* mutants (F) on Parafilm calculated using ImageJ pixel density analysis. (G and H) Sizes of infection cushions formed by Δ*SsEmp24* mutants (G) and Δ*SsErv25* mutants (H) calculated using ImageJ software. Data were analyzed by one-way ANOVA, and error bars represent the SD. *, *P < *0.01 in the variance analysis (*n* = 4, three independent experiments).

Infection cushions play an essential role in the pathogenicity of *S. sclerotiorum* (5). Thus, the infection cushion formation process of mutants on Parafilm and rapeseed leaves was investigated to analyze the reason for defective pathogenicity (Fig. 6). Δ*SsEmp24* mutants and Δ*SsErv25* mutants formed few small infection cushions on Parafilm and rapeseed leaves, in comparison to the complex infection cushions produced by the wild type and complementary transformants (Fig. 6A to C). Notably, the hyphal tips of infection cushions formed by Δ*SsEmp24* mutants and Δ*SsErv25* mutants failed to produce new infection cushions, which may be the reason why Δ*SsEmp24* mutants and Δ*SsErv25* mutants infection cushions were smaller (Fig. 6D). However, both Δ*SsEmp24* mutants and Δ*SsErv25* mutants still own the ability to form infection cushions, suggesting that defective infection cushion formation is not the only factor of limited pathogenicity in Δ*SsEmp24* mutants and Δ*SsErv25* mutants.

*S. sclerotiorum* produces copious acid, and the low pH contributes to establishing the optimum conditions for growth, reproduction, and pathogenicity of the fungus (33). Acid accumulation of Δ*SsEmp24* mutants and Δ*SsErv25* mutants was assayed on potato dextrose agar (PDA) medium with bromophenol blue, and a yellow color was observed, suggesting that Δ*SsEmp24* mutants and Δ*SsErv25* mutants could secret acid. However, acid accumulation of Δ*SsEmp24* mutants was much slower and less than that of the wild-type strain, but no significant difference was noted between Δ*SsErv25* mutants and wild-type strain (Fig. S4). A similar result was observed when strains were cultured in a potato dextrose broth (PDB) medium. The pH of the fermentation broth of the Δ*SsEmp24* mutants was significantly higher than that of the wild type and Δ*SsErv25* mutants during the early incubation stage (Fig. S4C).

10.1128/mBio.03173-21.4 FIG S4 Acid compound production assay. (A and B) Qualitative determination of acid produced by strains on PDA containing bromophenol blue dye as an ambient pH indicator. The presence of yellow color during hyphal extension indicates acid production. The wild type (WT) and complementary transformants (24Com and 25Com) were used as controls. (C) The ambient pH of fermentation broth was tested daily until 7 dpi. Data were analyzed by two-way ANOVA, and error bars represent the SD. *, *P < *0.01 in the variance analysis (*n* = 4, three independent experiments). Download FIG S4, TIF file, 2.8 MB. Copyright © 2021 Xie et al. 2021 Xie et al. https://creativecommons.org/licenses/by/4.0/ This content is distributed under the terms of the Creative Commons Attribution 4.0 International license.

### SsEmp24 and SsErv25 interact in the ER and nuclear envelope.

**FIG 7:**
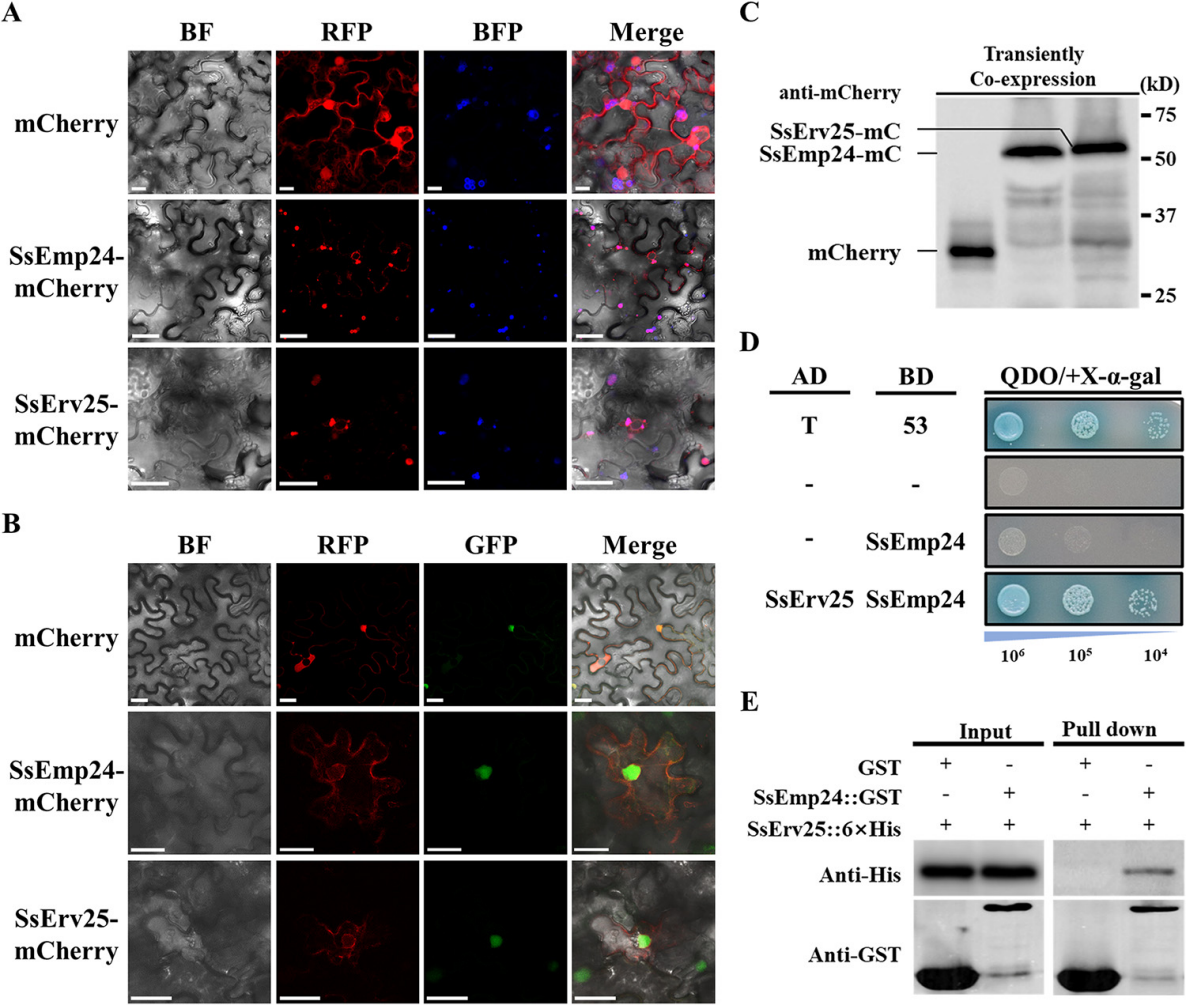
Protein localization and interaction assays. (A and B) Subcellular localization of SsEmp24 and SsErv25 in N. benthamiana. mCherry, SsEmp24-mCherry, and mCherry-SsErv25 were transiently coexpressed with CNX1-BFP (A) and GFP-SV40 (B) in N. benthamiana leaves. Images were captured under a CLSM at 48 h after coexpression. Scale bar, 25 μm. (C) The accumulations of mCherry, SsEmp24-mCherry, and SsErv25-mCherry in transiently coexpressed proteins were detected with anti-mCherry by Western blotting. mC, mCherry. (D) Yeast-two hybrid assay to determine the interaction between SsEmp24 and SsErv25. pGADT7-T and pGBKT7-53 were used as positive controls. (E) Confirmation of the interaction between SsEmp24 and SsErv25 by GST-pull down assay. The interaction was visualized by Western blotting with anti-His and anti-GST antibodies.

The transmembrane region and cytosolic tail of SsEmp24 and SsErv25 suggest that they could localize on the cytosolic membrane. To analyze the subcellular localization of SsEmp24 and SsErv25, the calnexin 1 protein-blue fluorescent protein (CNX1-BFP) that locates in the ER and the green fluorescent protein-simian virus 40 (GFP-SV40) that locates in the nucleus were employed as marker proteins, and mCherry that locates in the cytoplasm was used as a control. SsEmp24-mCherry or SsErv25-mCherry fusion protein was transiently coexpressed with CNX1-BFP or GFP-SV40 in Nicotiana benthamiana leaves. The results suggested that both SsEmp24-mCherry and SsErv25-mCherry were detected in the ER and nuclear envelope (Fig. 7A and B), and the accumulation and integrity of SsEmp24 and SsErv25 were confirmed by Western blot analysis (Fig. 7C).

Emp24p and Erv25p depend on each other and could form a protein complex in S. cerevisiae (25). The identical subcellular localization of SsEmp24 and SsErv25 in the ER and nuclear envelope (Fig. 7A and B) together with the yeast two-hybrid and pull down assays (Fig. 7D and E) provided strong evidence that SsEmp24 and SsErv25 interacted. Collectively, these observations suggested that SsEmp24 and SsErv25 interact and cooperate in the early secretory pathway to coregulate *S. sclerotiorum* morphogenesis and pathogenicity.

### SsEmp24 and SsErv25 affect the secretion of specific proteins.

**FIG 8:**
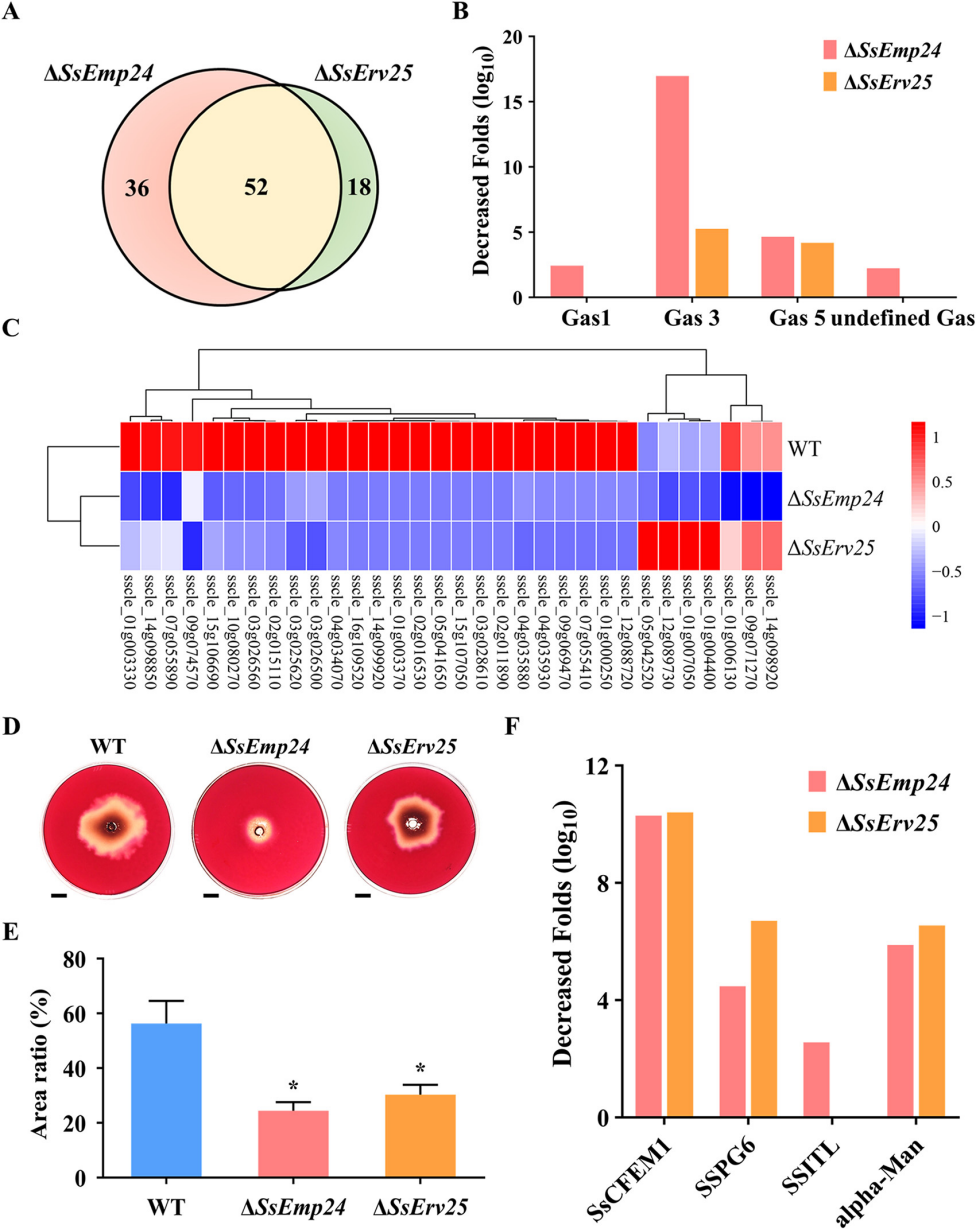
Secretory proteome analysis. (A) Venn diagram of the decreased proteins in Δ*SsEmp24* mutant and Δ*SsErv25* mutant. (B) Decreased fold (log_10_) in protein abundance in Δ*SsEmp24* mutant and Δ*SsErv25* mutant. Gas1, 1,3-beta-glucanosyltransferase Gas1 (accession number APA14203.1); Gas3, 1,3-beta-glucanosyltransferase Gas3 (accession number APA08637.1); Gas5, 1,3-beta-glucanosyltransferase Gas5 (accession number APA16182.1); undefined Gas, 1,3-beta-glucanosyltransferase (accession number APA09482.1). (C) Protein abundance of 32 glycoside hydrolases in the wild type (WT), Δ*SsEmp24* mutant, and Δ*SsErv25* mutant. Abundance is represented by color. High abundance is displayed in red, and low abundance is displayed in blue. (D) Cellulase secretion assay of the wild type (WT), Δ*SsEmp24* mutant, and Δ*SsErv25* mutant was conducted on cellulose medium. At 2 days after inoculation, the plates were treated with Congo red. A transparent circle indicates the area of cellulases secreted by the individual strain. Scale bar, 1 cm. (E) The ratio of cellulase secretion area to colony area in the wild type (WT), Δ*SsEmp24* mutant, and Δ*SsErv25* mutant. Data were analyzed by one-way ANOVA, and error bars represent the SD. *, *P < *0.01 in the variance analysis (*n* = 4, three independent experiments). (F) Decreased fold in protein abundance in Δ*SsEmp24* mutant and Δ*SsErv25* mutant. SsCFEM1, CFEM domain-containing protein in *S. sclerotiorum* (accession number APA10258.1); SSPG6, polygalacturonase SSPG6 (accession number APA14102.1); SSITL, integrin-like protein SSITL (accession number APA12080.1); alpha-Man, alpha-1,2-mannosidase (accession number APA07886.1).

Since *SsEmp24* and *SsErv25* were predicted to be involved in the early secretory pathway, secreted proteins in *S. sclerotiorum* were extracted and analyzed by mass spectrometry to clarify whether the secreted proteins were sorted by SsEmp24 and SsErv25. Principal-component analysis showed a relatively deviating sample in the wild type, which was removed from further analysis (Fig. S5A). A total of 244 proteins were detected in the secretory proteome (Table S3). Among them, 198 proteins containing a signal peptide with 132 unclassified secreted proteins, 43 GPI-APs, and 23 membrane proteins were secreted mainly depending on the conventional ER-to-Golgi secretory pathway (Fig. S5B). The abundance of 106 proteins significantly decreased (by >2-fold) in the Δ*SsEmp24* mutant or Δ*SsErv25* mutant compared with the wild type, including 36 and 18 proteins specifically decreased in Δ*SsEmp24* mutant or Δ*SsErv25* mutant, respectively (Fig. 8A; Table S4). Meanwhile, 52 proteins significantly decreased in both Δ*SsEmp24* mutant and Δ*SsErv25* mutant, including 28 secreted proteins, 8 GPI-APs, 3 membrane proteins, and 13 other proteins (Fig. S5C). Interestingly, 59 depleted proteins were annotated as extracellular hydrolase enzymes, with 32 proteins belonging to the glycoside hydrolase (GH) family, including cellulases, pectin lyases, and proteases (Fig. 8C; Table S4). Proteomic analysis and Congo red staining suggested that cellulase secretion in Δ*SsEmp24* mutant and Δ*SsErv25* mutant was compromised (Fig. 8C to E). Three aspartyl proteases (sscle_02g022040, sscle_03g025560, sscle_10g075920) were significantly decreased in Δ*SsEmp24* mutant (Table S4). Notably, the abundance of polygalacturonase SSPG6 (sscle_12g088720) decreased by ∼22- and ∼104-fold in Δ*SsEmp24* mutant and Δ*SsErv25* mutant, respectively (Fig. 8F). This is additional evidence that the secretion of hydrolase enzymes involved in the pathogenicity of *S. sclerotiorum* was affected by *SsEmp24* and *SsErv25* deletion. Furthermore, decreased secretion of effectors was also detected by proteomic analysis in Δ*SsEmp24* mutant and Δ*SsErv25* mutant compared with wild-type *S. sclerotiorum*. The effector SSITL (Sclerotinia sclerotiorum integrin-like protein; sscle_08g068500) that targets the calcium-sensing receptor in chloroplasts to interfere with the plant salicylic acid signaling pathway decreased by ∼5.9-fold in Δ*SsEmp24* mutant (Fig. 8F) (34, 35). Moreover, the secretion level of a putative alpha-mannosidase (sscle_02g016530), whose homologous protein has proved to be an effector in Podosphaera xanthii (36), was decreased by ∼24- and ∼8-fold in the Δ*SsEmp24* mutant and Δ*SsErv25* mutant, respectively (Fig. 8F).

10.1128/mBio.03173-21.5 FIG S5 Secretory proteome analysis. (A) Principal-component analysis (PCA) of secretory proteomes from the wild type (WT), Δ*SsEmp24* mutant (24KO), and Δ*SsErv25* mutant (25KO). (B) Number of proteins identified in secretory proteomes. (C) Number and classification of decreased proteins in Δ*SsEmp24* mutant, Δ*SsErv25* mutant, and both Δ*SsEmp24* mutant and Δ*SsErv25* mutant compared with the wild-type strain. Download FIG S5, TIF file, 0.4 MB. Copyright © 2021 Xie et al. 2021 Xie et al. https://creativecommons.org/licenses/by/4.0/ This content is distributed under the terms of the Creative Commons Attribution 4.0 International license.

10.1128/mBio.03173-21.9 TABLE S3 Detailed information about the secretory proteome that was detected in the wild type, Δ*SsEmp24* mutant, and Δ*SsErv25* mutant. Download Table S3, XLSX file, 0.06 MB. Copyright © 2021 Xie et al. 2021 Xie et al. https://creativecommons.org/licenses/by/4.0/ This content is distributed under the terms of the Creative Commons Attribution 4.0 International license.

10.1128/mBio.03173-21.10 TABLE S4 Predicted functional annotation of decreased proteins in Δ*SsEmp24* mutant and Δ*SsErv25* mutant, compared with the wild type. Download Table S4, XLSX file, 0.02 MB. Copyright © 2021 Xie et al. 2021 Xie et al. https://creativecommons.org/licenses/by/4.0/ This content is distributed under the terms of the Creative Commons Attribution 4.0 International license.

**FIG 9:**
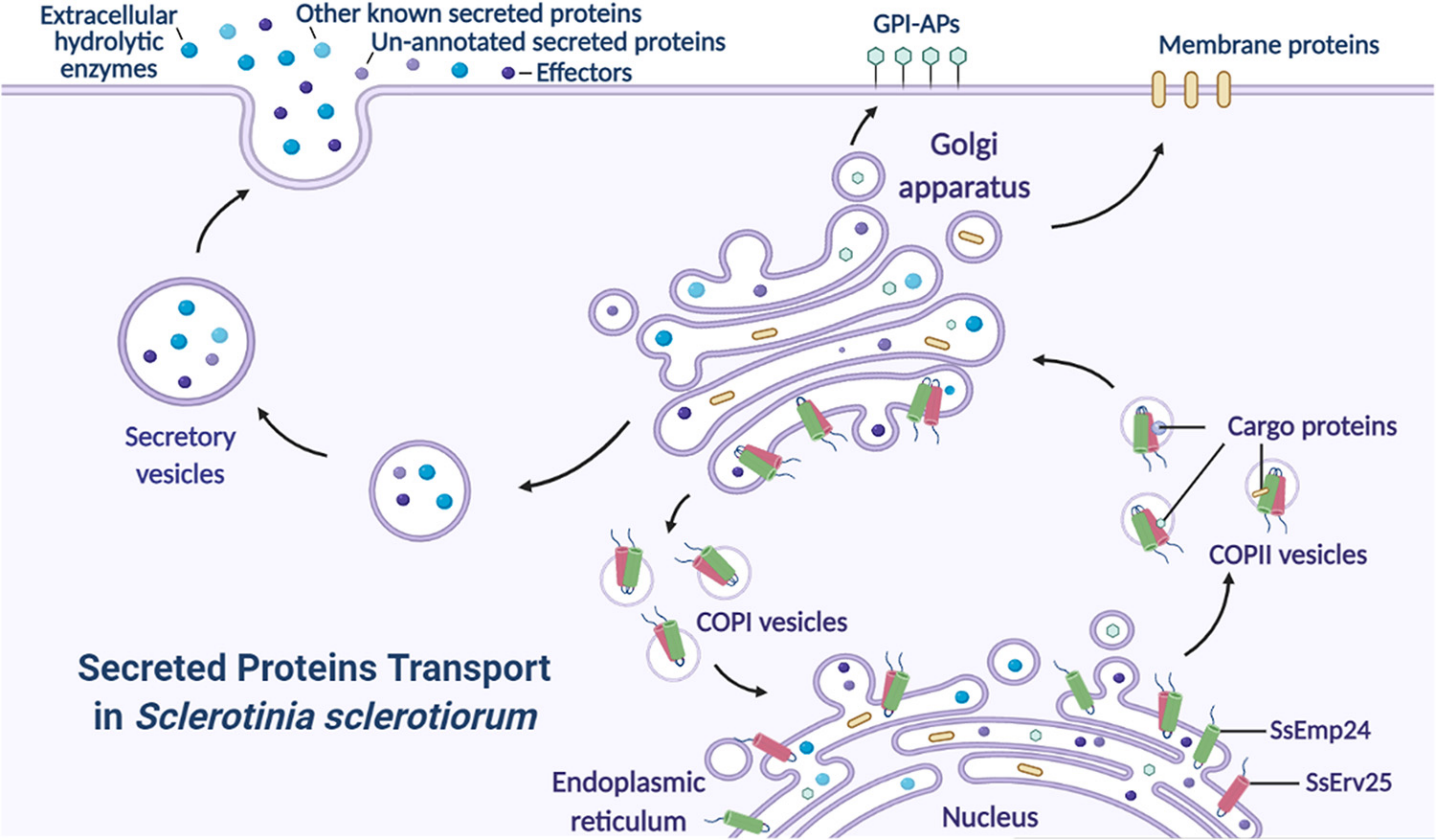
Schematic view of SsEmp24 and SsErv25 mediating the early secretory pathway to regulate the development and pathogenicity of *S. sclerotiorum*. SsEmp24/SsErv25 complexes are assembled into the vesicles as cargo receptors and cycle between the ER and the Golgi apparatus carrying specific cargo proteins. GPI-anchored proteins (GPI-APs), membrane proteins, and the secreted proteins, including extracellular hydrolase enzymes and the potential effectors, are translocated into the Golgi apparatus and then secreted extracellularly via secretory vesicles to participate in the pathogenic process of *S. sclerotiorum*. This figure was created in BioRender (https://biorender.com).

In summary, SsEmp24 interacts with SsErv25 to form a p24 protein complex. They act as cargo receptors to accept and carry specific cargo proteins, including secreted proteins, GPI-APs, and membrane proteins. Interestingly, secreted proteins involved in *S. sclerotiorum* pathogenicity, like extracellular hydrolase enzymes and effectors, are also cargos of p24 proteins, which are translocated to the Golgi apparatus, secreted extracellularly, and then participate in the fungal pathogenic process. Unloaded p24 proteins are translocated back to the ER by the Golgi apparatus in preparation for a new round of cargo transport (Fig. 9).

## DISCUSSION

Proper secretion of certain proteins is critical for the reproduction and pathogenicity of plant fungal pathogens, especially some known as effectors (37–39). More than one-third of newly synthesized proteins are targeted to the early secretory pathway (11). Heteromeric p24 protein complexes in S. cerevisiae are incorporated into COP vesicle biogenesis and cycling in the secretory pathway, which regulates the selection and transport of specific cargos (12, 13, 22, 40). However, the functions of p24 proteins in filamentous fungi were scarcely known prior to this study.

Eight p24 proteins identified in S. cerevisiae are divided into four subfamilies: p24α (Erp1p, Erp5p, and Erp6p), p24β (Emp24p), p24γ (Erp2p, Erp3p, and Erp4p), and p24δ (Erv25p). Meanwhile, eight to ten p24 proteins are found in mammals, while *A. thaliana* has twelve p24 proteins, lacking p24α and p24γ members (Fig. 1D). In contrast, only four p24 proteins were found in the yeast species Schizosaccharomyces pombe and Candida albicans and in the filamentous fungi R. solani, A. nidulans, *M. oryzae*, F. graminearum, *B. cinerea*, and *S. sclerotiorum* (Fig. 1D). The variable numbers of p24 proteins indicate that the function of p24 proteins may differ between species.

Disruption of certain proteins involved in the conventional ER-to-Golgi secretory pathway could affect effector secretion, morphogenesis, and pathogenicity in phytopathogenic fungi (41–43). Apoplastic effector secretion relies on the conventional fungal ER-to-Golgi secretory pathway in *M. oryzae*. Apoplastic effector secretion was blocked and apoplastic effectors were retained in the hyphal ER, when *M. oryzae* invasive hyphae were exposed to brefeldin A, which inhibits conventional ER-to-Golgi secretory pathway in fungi (38). However, whether the p24 proteins link the early secretory pathway and pathogenicity in phytopathogenic fungi is unknown. This study found that p24 proteins may serve as cargo receptors to mediate the secretion of proteins, including the pathogenicity-associated effectors and hydrolase enzymes. For instance, the hydrolase enzymes cellulase, aspartyl protease, and polygalacturonase are associated with virulence of *S. sclerotiorum* (35, 36), but the secretion of these enzymes was decreased in the Δ*SsEmp24* mutant and Δ*SsErv25* mutant. Even though the p24 mutants were able to penetrate the plant surface successfully, the reduction of these hydrolase enzymes may lead to attenuation of the ability to degrade the host cell wall, and the reduction of effectors may cause a decreased ability to interfere with the host immune response, thus limiting the expansion of the lesion. This prediction was consistent with the result of pathogenicity analysis on wounded leaves, that lesion size caused by p24 gene deletion mutants on intact and wounded leaves has no significant difference.

Emp24p, Erv25p, Erp1p, and Erp2p often form a polymeric complex at ∼130,000 molecules per cell in S. cerevisiae (44, 45). Similar to their S. cerevisiae counterparts, SsEmp24 interacted with SsErv25 and colocalized predominantly in the ER or nuclear envelope. However, further study is required to determine whether the four p24 proteins form tetrameric complexes in *S. sclerotiorum*. The S. cerevisiae strain p24Δ8 that lacks all eight members of p24 proteins has growth similar to that of the wild type (44). In contrast, this study revealed that two p24 proteins, SsEmp24 and SsErv25, interact together and are involved in reproduction and pathogenicity in the phytopathogenic fungus *S. sclerotiorum*. Likewise, loss of p24 proteins also affected hyphal growth in the filamentous Trichoderma reesei, Penicillium decumbens, and Penicillium oxalicum (46, 47). This suggests that the function of p24 proteins in filamentous fungi and yeast species might be divergent. S. cerevisiae strain p24Δ8 showed a delay of invertase and Gas1p ER-to-Golgi transport which was identical to that seen in a single Δ*emp24* deletion strain (44). Similarly, this study found that SsEmp24 played a more crucial role than SsErv25, since there were significantly smaller lesions on leaves caused by Δ*SsEmp24* mutant than that caused by Δ*SsErv25* mutant, and Δ*SsEmp24* mutant completely lost the ability to produce sclerotia. Thus, although SsEmp24 and SsErv25 interact with each other, we cannot exclude the possibility that certain functions of SsEmp24 and SsErv25 are independent of protein dimerization, which may be one reason for the incompletely consistent phenotype of the Δ*SsEmp24* mutant and Δ*SsErv25* mutant. The GOLD domain is predicted to mediate diverse protein-protein interactions related to secretion or protein sorting (48). The loops of the GOLD domain differ between filamentous fungi and yeast species, possibly leading to a distinct cargo-carrying capacity of p24 proteins and resulting in different p24 mutant phenotypes in filamentous fungi and yeast species. In addition, the cytoplasmic tail sequences of Emp24p and Erv25p have distinct roles in transport between the ER and Golgi apparatus, by binding subunits of the COPII coat and promoting export from the ER; however, only the Erv25p tail sequence binds COPI and is responsible for returning this complex to the ER in S. cerevisiae (21). Therefore, we speculate that the different loops of the GOLD domains and cytoplasmic tail sequences of SsEmp24 and SsErv25 are possibly another reason for the different phenotypes of the Δ*SsEmp24* mutant and Δ*SsErv25* mutant. Previous work showed that the S. cerevisiae homolog of Ss-Caf1 facilitates secretion by sorting cargo proteins in the early secretory pathway (14, 15). Moreover, *SsEmp24* and *SsErv25* expression levels in the *Ss-caf1* disruption mutant significantly decreased compared with that in the wild type. Therefore, the relationship of Ss-Caf1 and p24 proteins in the early secretory pathway is worth further exploring in *S. sclerotiorum*.

p24 proteins are proposed to function as cargo receptors for the transport of various proteins, including GPI-APs, GPIG-protein-coupled receptors, Wnt proteins, Toll-like receptors, and a putative myrosinase-associated protein, GLL23 (49). The ER exit of GPI-APs is promoted by p24 proteins, and strains lacking Emp24p or Erv25p have delayed the Gas1p transport in S. cerevisiae (12, 20, 24, 44). In the present study, the abundance of 8 GPI-APs decreased in the Δ*SsEmp24* mutant or Δ*SsErv25* mutant. For instance, the abundance of predicted GPI-AP Gas1p (sscle_12g089730) secretion in *S*. *sclerotiorum* significantly decreased by ∼5.4-fold in Δ*SsEmp24* mutant (Fig. 8B; see Table S4 in the supplemental material). Therefore, the GPI-AP transport function of p24 proteins may be conserved in fungi, and Gas proteins might be the specific cargos of the p24 complex. A GPI-AP (sscle_06g050280) containing the CFEM (common in several fungal extracellular membrane proteins) domain was barely detected in both the Δ*SsEmp24* mutant and Δ*SsErv25* mutant (Table S4), and its homologous protein BcCFEM1 in *B. cinerea* (BC1G_15201) contributes to virulence (50). This suggested that the decreased GPI-APs may be one reason for the defect in pathogenicity of Δ*SsEmp24* mutant and Δ*SsErv25* mutant. The GPI-APs are secreted and attached to the surface of cells and have diverse functions, ranging from growth, cell wall biosynthesis, cell adhesion, and plasmodesmatal transport (20, 40). Hence, defective GPI-AP secretion in Δ*SsEmp24* mutant and Δ*SsErv25* mutant may be the possible reason for their abnormal growth.

In summary, we investigated two p24 proteins in *S. sclerotiorum*, SsEmp24 and SsErv25, that formed a complex and were involved in vegetative growth, sclerotial formation, infection cushion formation, and pathogenicity via regulation of proper protein secretion in *S. sclerotiorum*. SsEmp24 may become a seminal tool for studying the function of components of the early secretory pathway, since Δ*SsEmp24* mutant had a more severe phenotype than Δ*SsErv25* mutant. This study contributes to a better understanding of the role of p24 proteins and the secretory pathway in filamentous phytopathogenic fungi, provides clues for screening pathogenic factors, and inspires new potential strategies to control fungal disease.

## MATERIALS AND METHODS

### Fungal strains and plant cultivation.

The *S*. *sclerotiorum* wild-type strain Sunf-M (WT) was used to generate gene knockout strains, Δ*SsEmp24* mutants (24KO-1, 24KO-2, and 24KO-3) and Δ*SsErv25* mutants (25KO-1 and 25KO-2), and their corresponding complementary strains, Δ*SsEmp24*-Com strain (24Com) and Δ*SsErv25*-Com strain (25Com). The wild-type strain was routinely cultured on potato dextrose agar (PDA) plates at 20°C. All transformants were maintained on PDA supplemented with 100 μg/ml hygromycin B (Roche, Switzerland) or 100 μg/ml G418 sulfate (Sigma, USA). N. benthamiana, rapeseed, and soybean were grown at 23°C (16-h-light/8-h-dark cycle, 70% humidity) in a greenhouse.

### Multiple alignment, phylogenetical analysis, and conserved domain identification.

The sequences referenced in this study were retrieved from the National Center for Biotechnology Information GenBank database (http://www.ncbi.nlm.nih.gov/), and accession numbers for all genes or proteins are listed in Table S1 in the supplemental material. The amino acid sequences of p24 family proteins from S. cerevisiae were used as query sequences to conduct BLASTP, and members of the p24 family in *S. sclerotiorum* were screened out. The SignalP-5.0 server (https://services.healthtech.dtu.dk/service.php?SignalP-5.0/), TMHMM server v.2.0 (https://services.healthtech.dtu.dk/service.php?TMHMM-2.0/), and TOPCONS web server (https://topcons.net/) were used to predict signal peptides and transmembrane regions in p24 proteins. GPI-SOM (http://gpi.unibe.ch/) was used with SignalP in genome-wide surveys for GPI-anchored proteins. The conserved functional domains of p24 proteins were predicted using ScanProsite (https://prosite.expasy.org/scanprosite/) and InterPro (https://www.ebi.ac.uk/interpro/). Protein modeling was performed with the I-TASSER server (https://zhanglab.ccmb.med.umich.edu/I-TASSER/), and models were compared using Chimera (http://www.rbvi.ucsf.edu/chimera/).

For phylogenetic analysis, multiple alignment was performed with p24 proteins in *S. sclerotiorum* and their homologs using the MUSCLE program of MEGA 7 (https://www.megasoftware.net/) with default parameters. Thereafter, the phylogenetic tree was generated using the neighbor-joining method in MEGA 7 with 1,000 bootstrap replicates and exported into the Interactive Tree of Life (http://itol.embl.de) for further annotation.

### RNA extraction and RT-qPCR analysis.

To profile the expression of p24 genes in infection cushion formation stages, the wild-type strain was inoculated on Parafilm for 4 h and 24 h, and the *Ss-caf1* gene disruption mutant was inoculated on Parafilm for 4 h. To profile the expression of p24 genes in the infection process, the wild-type strain was inoculated on intact rapeseed leaves for 4 h, 8 h, and 24 h. The wild-type strain inoculated for 0 h was used as a control, and tissues of hypha were collected for RNA preparation and RT-qPCR. To profile the expression of p24 genes in infection cushions, the wild-type strain was inoculated on cellophane or Parafilm for 24 h, and tissues of hypha or infection cushions were collected for RNA preparation and RT-qPCR.

Total RNA was extracted with the RNAiso Plus kit (TaKaRa, China), and the PrimeScript RT reagent kit with gDNA eraser (perfect real time; TaKaRa, China) was used for cDNA synthesis. Gene expression abundance of the target gene was quantified by the ViiA 7 real-time PCR system (Applied Biosystems, USA) using the TB Green Premix Ex Taq II (Tli RNaseH Plus; TaKaRa, China). Primer pairs used for RT-qPCR are listed in Table S2, and the primer pairs qEmp24-F/qEmp24-R, qErv25-F/qErv25-R, qSsErp1-F/qSsErp1-R, and qSsErp3-F/qSsErp3-R were used to evaluate expression levels of the *SsEmp24*, *SsErv25*, *SsErp1*, and *SsErp3* genes, respectively. The fold changes in gene expression were calculated compared to the control by use of the 2^−ΔΔ^*^CT^* method, and data were normalized against the housekeeping gene *actin* as an endogenous reference. This experiment was repeated with RNA from at least three biological replicates, with each treatment set having four replicates.

10.1128/mBio.03173-21.8 TABLE S2 Detailed information about primers used in this study. Download Table S2, XLSX file, 0.01 MB. Copyright © 2021 Xie et al. 2021 Xie et al. https://creativecommons.org/licenses/by/4.0/ This content is distributed under the terms of the Creative Commons Attribution 4.0 International license.

### *SsEmp24* and *SsErv25* gene replacement.

The strategy based on the split-marker approach was used to obtain *SsEmp24* and *SsErv25* gene knockout strains (Fig. S6). Approximately 1.5-kb upstream flanking sequences of genes *SsEmp24* and *SsErv25* were cloned with primer pairs 24-5′-F/24-5′-R and 25-5′-F/25-5′-R, respectively (Table S2). Approximately 1.5-kb downstream flanking sequences of genes *SsEmp24* and *SsErv25* were amplified using primer pairs 24-3′-F/24-3′-R and 25-3′-F/25-3′-R, respectively. The 2.1-kb hygromycin phosphotransferase (*hph*) gene from vector pUCH18 was used as a template to clone the front sequences of *hph*, termed HP, and the rear sequences of *hph*, termed PH. Thereafter, overlapping PCR was used to fuse fragments. The primers 24-5′-F and hp-R were used to fuse the upstream flanking sequence of *SsEmp24* with HP, and primers ph-F and 24-5′-R were used to fuse the downstream flanking sequence of *SsEmp24* with PH. The upstream flanking sequence of *SsErv25* was fused with HP using PCR with primers 25-5′-F and hp-R, and the downstream flanking sequence of *SsErv25* was fused with PH using primers ph-F and 25-5′-R. The overlap between the two truncated *hph* gene fragments was 704 bp. The two overlapping fragments were concurrently transformed into the protoplasts of the *S. sclerotiorum* wild-type strain, with reference to the method described by Rollins (51). Hygromycin-resistant transformants were selected in regeneration agar medium with 100 μg/ml hygromycin B and screened using PCR. As described in Fig. S6, primer pairs 24Up-F/Up-R and Down-F/24-Down-R were used to screen *SsEmp24* gene knockout transformants, and 25Up-F/Up-R and Down-F/25-Down-R were used to identify *SsErv25* gene knockout transformants. Gene knockout transformants were further verified using Southern blotting. Briefly, the enzyme BamHI or XbaI was used to digest the genomic DNA of each transformant. A 499-bp *hph* gene fragment generated by PCR amplification with primer pair hph-probe-F/hph-probe-R was used as a probe (probe 2) to analyze *hph* gene copies in transformants. The downstream fragment of *SsEmp24* and fragment of *SsErv25* were amplified with primer pairs Emp24-probe-F/Emp24-probe-R and Erv25-probe-F/Erv25-probe-R, respectively, and used as probes (probe 1 and probe 3) to analyze target gene copies in transformants. Probe labeling and hybridization were performed according to standard protocols with the AlkPhose Direct labeling and detection system (GE Health, USA) for Southern blotting.

10.1128/mBio.03173-21.6 FIG S6 Gene replacement construction based on the split-marker approach. (A) *SsEmp24* gene replacement construct containing the hygromycin phosphotransferase (*hph*) cassette in place of the *SsEmp24* gene. (B) *SsErv25* gene replacement construct containing the hygromycin phosphotransferase (*hph*) cassette in place of the *SsErv25* gene. Download FIG S6, TIF file, 0.5 MB. Copyright © 2021 Xie et al. 2021 Xie et al. https://creativecommons.org/licenses/by/4.0/ This content is distributed under the terms of the Creative Commons Attribution 4.0 International license.

### *SsEmp24* and *SsErv25* complementary transformants.

To obtain complementary transformants of *SsEmp24* and *SsErv25* gene knockout transformants, the full coding sequences of *SsEmp24* and *SsErv25* were amplified with primer pairs Emp24-OE-F/Emp24-OE-R and Erv25-OE-F/Erv25-OE-R (Table S2). The promoter of the *gpds* gene (GenBank accession number XM_001591123.1) in *S. sclerotiorum*, termed PgpdS, was cloned with primers PgpdS-F and PgpdS-R. Thereafter, fragments with the promoter PgpdS and target genes were generated by overlapping PCR with primer pairs PgpdS-F/Emp24-OE-R and PgpdS-F/Erv25-OE-R, respectively. Primers npt-F and npt-R were used to amplify the neomycin phosphotransferase gene (*npt*). The overlapping PCR fragment and *npt* fragment were cotransformed into protoplasts of target gene knockout transformants, with reference to the method described by Rollins (51). Complementary transformants were screened in regeneration agar medium with 100 μg/ml G418 sulfate. Primers npt-F and npt-R were used to confirm the *npt* gene in transformants. The expression of target genes *SsEmp24* and *SsErv25* in transformants was evaluated using RT-PCR with primer pairs qEmp24-F/qEmp24-R and qErv25-F/qErv25-R, respectively.

### Phenotypic characteristics.

To assess fungal growth characteristics, fresh hyphal plugs (5 mm in diameter) were inoculated on a PDA plate (9 cm in diameter), and the colony diameters were measured every 12 h for 3 days. The sclerotial formation on PDA was checked after 14 dpi, and sclerotia were collected and dried at 37°C for 2 weeks. The diameter per sclerotium was measured using ImageJ software (https://imagej.nih.gov/ij/), and the weight and number of sclerotia per plate were subsequently recorded. The experiment was repeated three times, with three replicates.

### Pathogenicity assays.

To test the pathogenicity of strains, rapeseed and soybean were used for the pathogenicity assay as described by Xiao et al. (5). Fresh hyphal plugs (5 mm in diameter) were inoculated on the detached leaves or living plants of rapeseed or soybean. The inoculated detached leaves or living plants were maintained at 90% relative humidity in a greenhouse. Leaves gently treated with sandpaper prior to inoculation were set as the wounded treatment, and the mycelial plugs were placed over the wounds. The wild-type strain was used as a control under the same conditions. The diameters of lesions on rapeseed leaves were recorded, and the areas of lesions on soybean leaves were counted using the ImageJ software. The experiment was repeated four times with four replicates.

### Infection cushion formation assay.

To induce infection cushion formation, a 5-mm fresh hyphal plug of each strain was inoculated on Parafilm-overlaid PDA or rapeseed leaves, and the plate or leaves were incubated at 20°C. The infection cushions formed on the Parafilm were stained with 0.05% trypan blue in a lactophenol solution (20% lactic acid, 20% phenol, 40% glycerol, and 20% water) for 1 h. Thereafter, the Parafilm was rinsed with water to remove the staining solution and placed in 50% glycerol on glass slides for observation with light microscopy. The number and size of the infection cushions were determined using ImageJ software. The experiment was repeated three times, with a set of four replicates.

The infection cushions formed on the rapeseed leaves were observed by scanning electron microscopy (SEM) as previously described (5). Rapeseed leaves from the edge of a lesion induced by the hyphal plug were cut into small pieces (3 by 3 mm), and 10 pieces of tissue for each sample were collected. All SEM samples were immersed in 2.5% (wt/vol) glutaraldehyde solution in sodium phosphate buffer (0.05 M, pH 7.0) at 4°C and vacuumed to immerse the tissues overnight. The tissues were then washed for 10 min three times in 0.05 M sodium phosphate buffer (pH 7.0) and dehydrated in a degraded ethanol series. After critical point drying and gold-coating in a sputter coater, the samples were observed for infection cushions under an SEM (JSM-6390/LV; NTC, Japan). The experiment was repeated three times, with a set of four replicates.

### Acid secretion assay.

To evaluate the acid product, fresh hyphal plugs of strains were cultured on PDA plates supplemented with bromophenol blue (1 mg/ml) at 20°C, and pictures were taken each day until 7 dpi. Strains were inoculated in potato dextrose broth (PDB) and incubated at 20°C with shaking at 150 rpm. The pH of cultures incubated for different days was tested daily using a pH meter (Mettler Toledo, Switzerland) to determine the acid production by strains. The assay was repeated three times with a set of four replicates.

### Cellulase secretion assay.

To evaluate the cellulase secretion, fresh hyphal plugs of strains were inoculated on plates containing 1.5% agar and 0.5% sodium carboxymethylcellulose as the substrate and incubated at 20°C for 2 days. Plates were stained for 20 min with 1% Congo red after incubation. The staining solution was washed with 1 M NaCl and 0.5% acetic acid and rinsed off with water immediately. A transparent or orange circle indicated the area of cellulases secreted by the individual strains. The area size of the transparent or orange circle and colony were counted using ImageJ software, and the ratio was calculated. The experiment was repeated three times, with a set of four replicates.

### Subcellular localization of SsEmp24 and SsErv25.

To clarify the localization of SsEmp24 and SsErv25, the vector pCNMC was used to transiently express proteins SsEmp24 and SsErv25 in N. benthamiana by an agroinfiltration method as previously described (52). The coding sequences of genes *SsEmp24* and *SsErv25* were amplified with primer pairs Emp24-CNMC-F/Emp24-CNMC-R and Erv25-CNMC-F/Erv25-CNMC-R (Table S2), respectively. The sequenced PCR products were digested with BglII/KpnI or BamHI/KpnI and subsequently ligated into the plasmid pCNMC to produce the vectors pCNMC-Emp24 and pCNMC-Erv25. The vector pCNCNX1-BFP, which can express a blue fluorescent protein (BFP) fused with the ER localization protein CNX1 (calnexin 1; NCBI accession number NP_200987.1), and the vector pCNGNLS, which includes an enhanced green fluorescent protein (eGFP) fused with the nuclear localization sequence (NLS) PKKKRKV of the SV40 large-T antigen, were used as marker proteins (53). All the expression vectors were transferred into Agrobacterium tumefaciens GV3101 by electroporation, respectively. Bacterial cultures containing expression vector (optical density at 600 nm [OD_600_], ∼0.6) were resuspended in infiltration solution (10 mM MgCl_2_, 10 mM 4-morpholineethanesulfonic acid, and 200 μM acetosyringone in deionized water). Bacterial cultures were mixed at a 1:1 ratio and infiltrated into N. benthamiana leaves using 1-ml needleless syringes for the coexpression. Transient expression was performed by coexpression of pCNMC-Emp24 or pCNMC-Erv25 with pCNCNX1-BFP or pCNGNLS, and the pCNMC coexpressed with pCNCNX1-BFP or pCNGNLS was used as the control treatment. To analyze the protein subcellular location, fluorescence in N. benthamiana leaves was monitored at 2 days post agroinfiltration and imaged directly using a confocal laser scanning microscope (CLSM). For CLSM (Leica, Germany) analysis, the excitation wavelengths were set to 405 nm for BFP, 488 nm for GFP, and 561 nm for mCherry. Transcription and expression of mCherry, SsEmp24-mCherry, and SsErv25-mCherry were measured via Western blotting with mCherry antibody (Proteintech, USA).

### Yeast two-hybrid and pull down assays for protein interaction verification.

The Matchmaker gold yeast two-hybrid (Y2H) system (Clontech, USA) was used to explore the interaction between SsEmp24 and SsErv25. For the Y2H assay, the coding sequences between the signal peptide and transmembrane region for genes *SsEmp24* (H^22^–R^170^) and *SsErv25* (L^24^–R^186^) were amplified from cDNA of *S. sclerotiorum* with primer pairs Emp24-BD-F/Emp24-BD-R and Erv25-AD-F/Erv25-AD-R (primers listed in Table S2). The PCR fragment for gene *SsEmp24* was digested by EcoRI and PstI and ligated into the Y2H vector pGBKT7 to generate the prey construct BD-Emp24. The fragment for gene *SsErv25* was digested with EcoRI and BamHI and introduced into Y2H vector pGADT7 to generate the bait construct AD-Erv25. The resulting bait and prey vectors were cotransformed in pairs into yeast strain Y2H Gold (Clontech, USA) according to the manufacturer’s instructions, and the frozen competent yeast cells were produced according to the method described by Gietz and Schiestl (54). The yeast transformant growth was analyzed on synthetic defined (SD)/-Trp-Leu medium (SD medium without Trp and Leu) and SD/-Trp-Leu-His-Ade medium (SD medium without Trp, Leu, His, and adenine hemisulfate) containing X-α-galactosidase (Clontech, USA) and Aureobasidin A (Clontech, USA).

The pull down assay was performed as described by Yang et al. (52). Briefly, the coding sequences between the signal peptide and transmembrane region for genes *SsEmp24* (H^22^–R^170^) and *SsErv25* (L^24^–R^186^) were cloned with primer pairs Emp24-GST-F/Emp24-GST-R and Erv25-His-F/Erv25-His-R. The PCR product of the gene *SsEmp24* was digested with EcoRI and SalI and ligated into plasmid pGEX-6P-1 to generate the vector pGEX-Emp24. The PCR product of the gene *SsErv25* was digested with BamHI and EcoRI and ligated into plasmid pET-28a to generate vector pET-Erv25. The plasmids pGEX-6P-1, pGEX-Emp24, and pET-Erv25 were expressed in Escherichia coli strain Rosetta (DE3) for 8 h at 20°C by IPTG (isopropyl-β-d-thiogalactopyranoside) (0.1 mM) induction to obtain purified proteins of glutathione *S*-transferase (GST), SsEmp24::GST, and SsErv25::6×His, respectively. An equal amount of GST or SsEmp24::GST sonicated lysates were mixed with high-affinity GST resin, and SsErv25::6×His was subsequently added to the mixture and incubated at 4°C overnight. Finally, bound proteins were eluted with fresh 10 mM glutathione elution buffer (10 mM glutathione reduced, 50 mM Tris-HCl, pH 8.0). The antibodies, anti-GST (Proteintech, USA) and anti-His (Proteintech, USA), were used to perform Western blot analysis to determine the interactions between proteins.

### Protein secretion assay.

To evaluate the secretory proteins affected by SsEmp24 and SsErv25, *S. sclerotiorum* strains were cultured in Czapek-Dox medium at 20°C with shaking at 150 rpm. Samples of each treatment in liquid-state fermentations were collected at 7 dpi and used to extract proteins for proteome analysis. The fermentations were filtered through a 0.45-μm sterile membrane to carry out the extracellular protein extraction. Purified secretome samples were frozen at −80°C and then concentrated overnight using a FreeZone 6-liter benchtop freeze dryer (Labconco). After thawing, samples were added to the ultrafiltration tube (10 kDa; Millipore, USA) and centrifuged for 20 min at 10,500 × *g* to discard the polysaccharides. The samples were collected and stored at −80°C until further analysis. Three biological repeats existed for each condition in the proteome.

The whole Sequential Windowed Acquisition of all Theoretical fragment ions (SWATH) analysis was submitted to a company (GeneCreate, Wuhan, China), and the mass spectrometry assay was conducted briefly as follows. One hundred micrograms of protein from each sample was digested by trypsin at 37°C for 12 to 16 h, desalted using C_18_ columns, and dried with a vacuum concentration meter. A high-performance liquid chromatography (HPLC) system (Thermo Scientific Dionex Ultimate 3000 BioRS) with a Welch C_18_ column (5 μm, 120 Å, 4.6 by 250 mm) was used for sample fractionation. Finally, collected fractions were combined into 10 fractions and dried by vacuum centrifugation. The data were collected using the TripleTOF 5600 and liquid chromatography/mass spectrometry (LC/MS) system (AB SCIEX, USA). Data-dependent acquisition (DDA) was followed by SWATH acquisition, where all samples were mixed and detected in DDA, and the resulting data were used as a library for the analysis of each sample by SWATH. Spectral library generation and SWATH data processing were performed using Skyline version 3.5 software. Fragment ion areas that belonged to one peptide were added to obtain a peptide’s abundance, and the total abundance of peptides for a given protein was determined to obtain the abundance of protein. To eliminate the random errors and sample bias, all the data among samples were normalized using the median normalization method.

Protein annotation was manually performed by combining information from NCBI gene descriptions, UniProt databases, and some previous articles. The SignalP-5.0 server, TMHMM server v.2.0, and TOPCONS web server were used to predict signal peptides and transmembrane regions in p24 proteins. GPI-SOM was used with SignalP in genome-wide surveys for GPI-anchored proteins. The conserved functional domains of p24 proteins were predicted using ScanProsite and InterPro. Heatmap plotting was performed using the R package pheatmap.

### Statistical analysis.

The data of different biological treatments were subjected to one-way analysis of variance (ANOVA) or two-way ANOVA, and statistical analyses were performed using Prism 8 (GraphPad Software, USA). The results of comparisons are presented as the mean ± standard deviation (SD), and the significant difference was evaluated at a *P *of <0.01.
