# Supplementary figures and images for: Early Secretory Pathway-Associated Proteins SsEmp24 and SsErv25 Are Involved in Morphogenesis and Pathogenicity in a Filamentous Phytopathogenic Fungus
Source: mBio. 2021 Dec 21;12(6):e03173-21. doi: 10.1128/mBio.03173-21 (PMC8689567; doi:10.1128/mBio.03173-21)

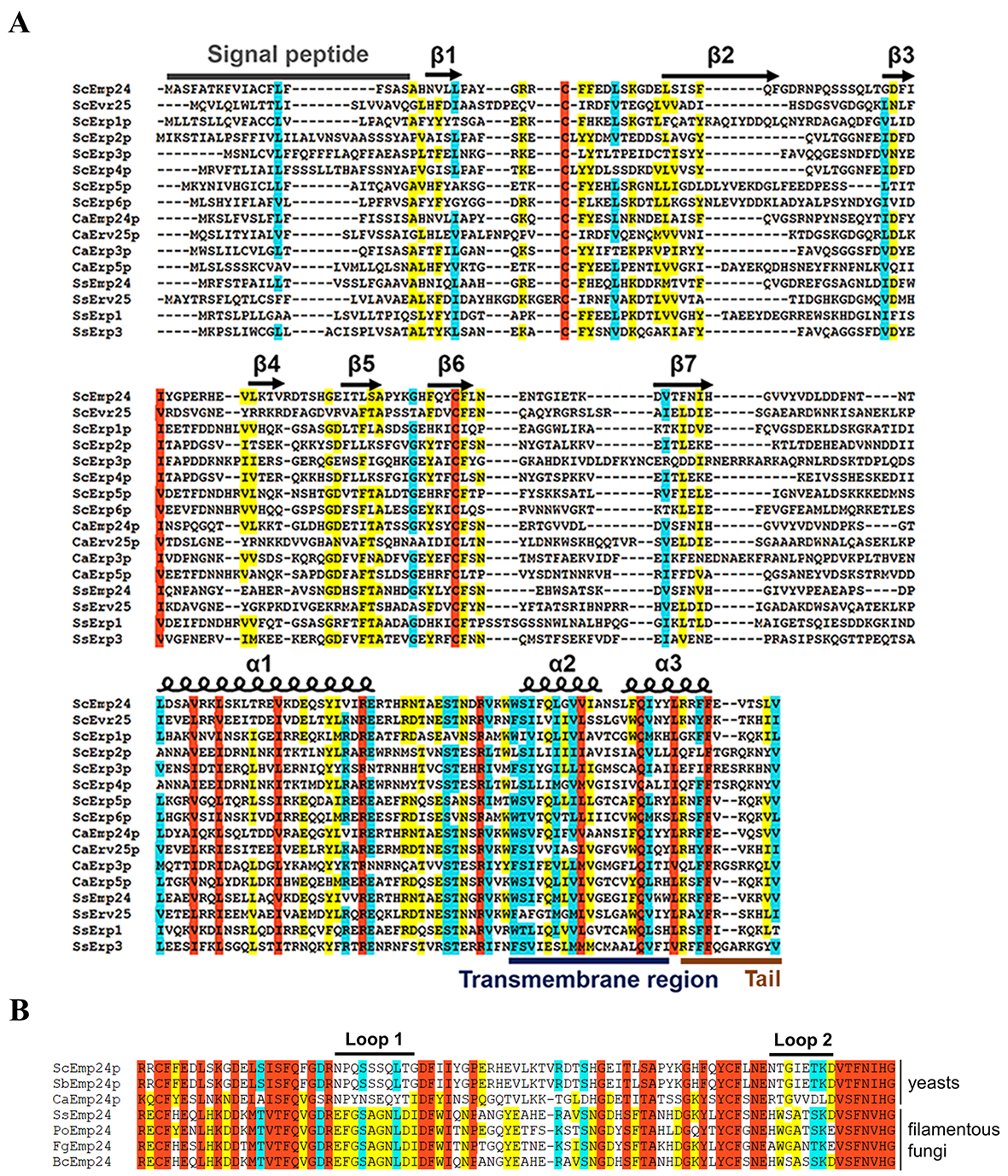

Supplement: FIG S1 [file mbio.03173-21-sf001.tif]

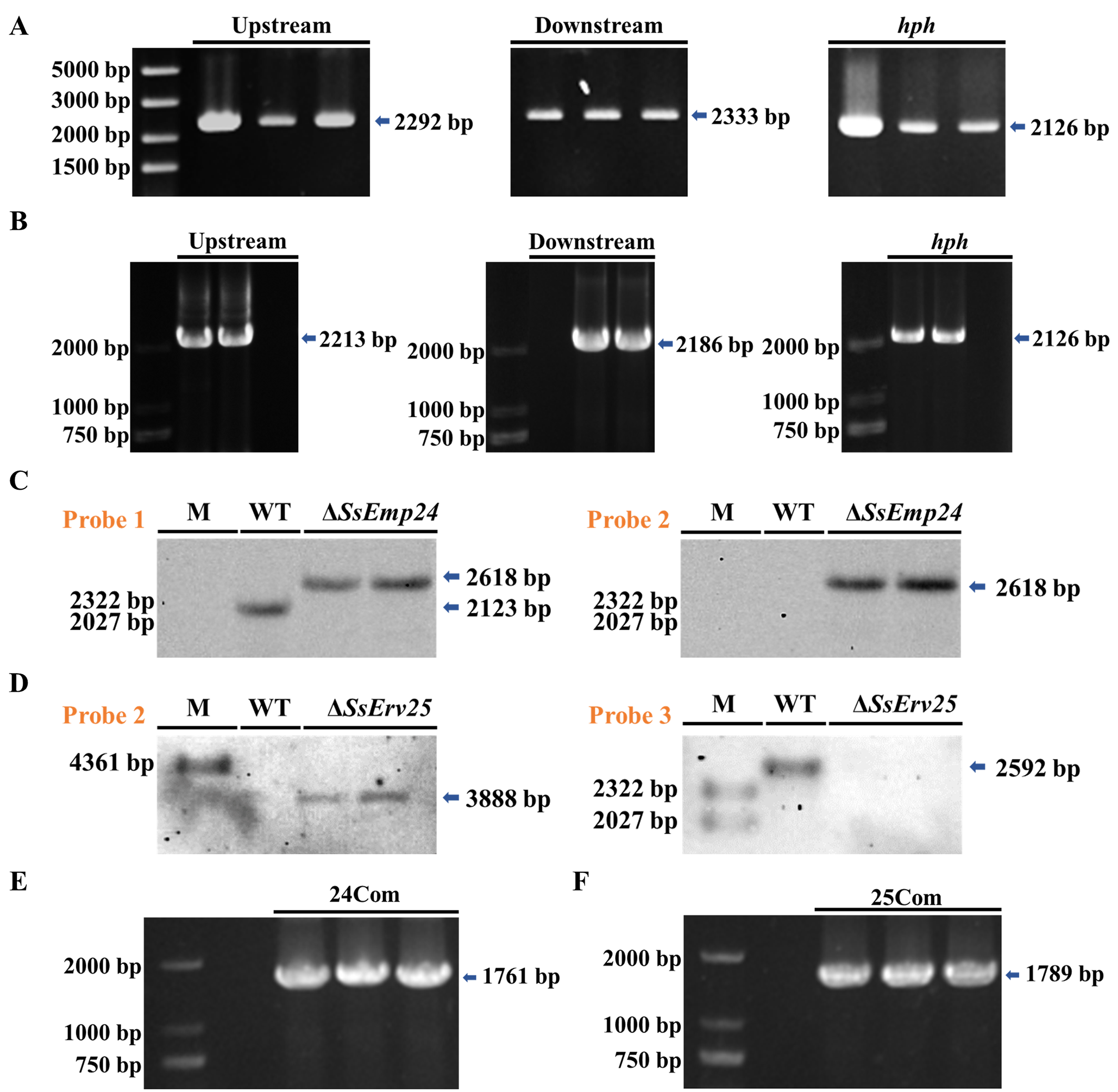

Supplement: FIG S2 [file mbio.03173-21-sf002.tif]

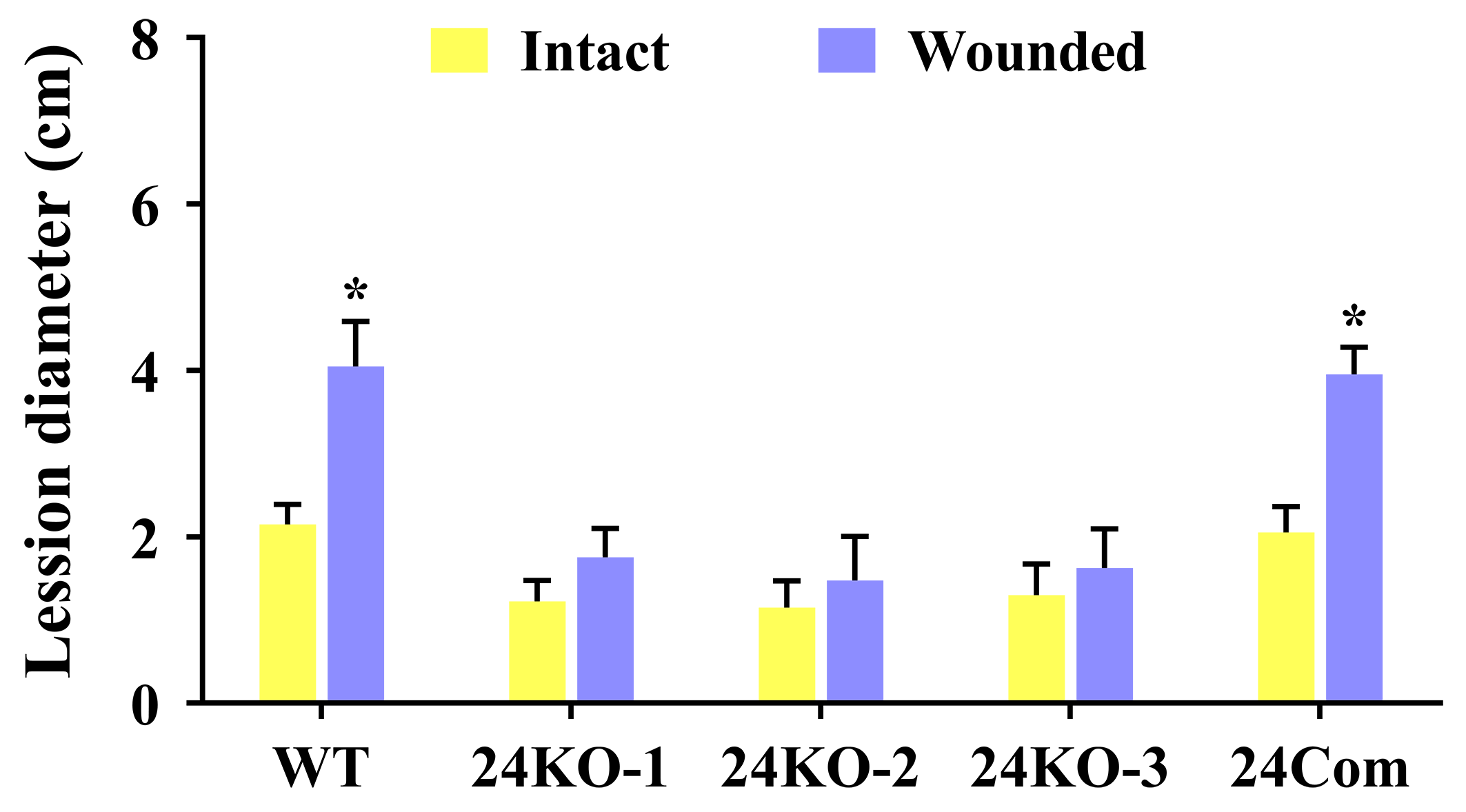

Supplement: FIG S3 [file mbio.03173-21-sf003.tif]

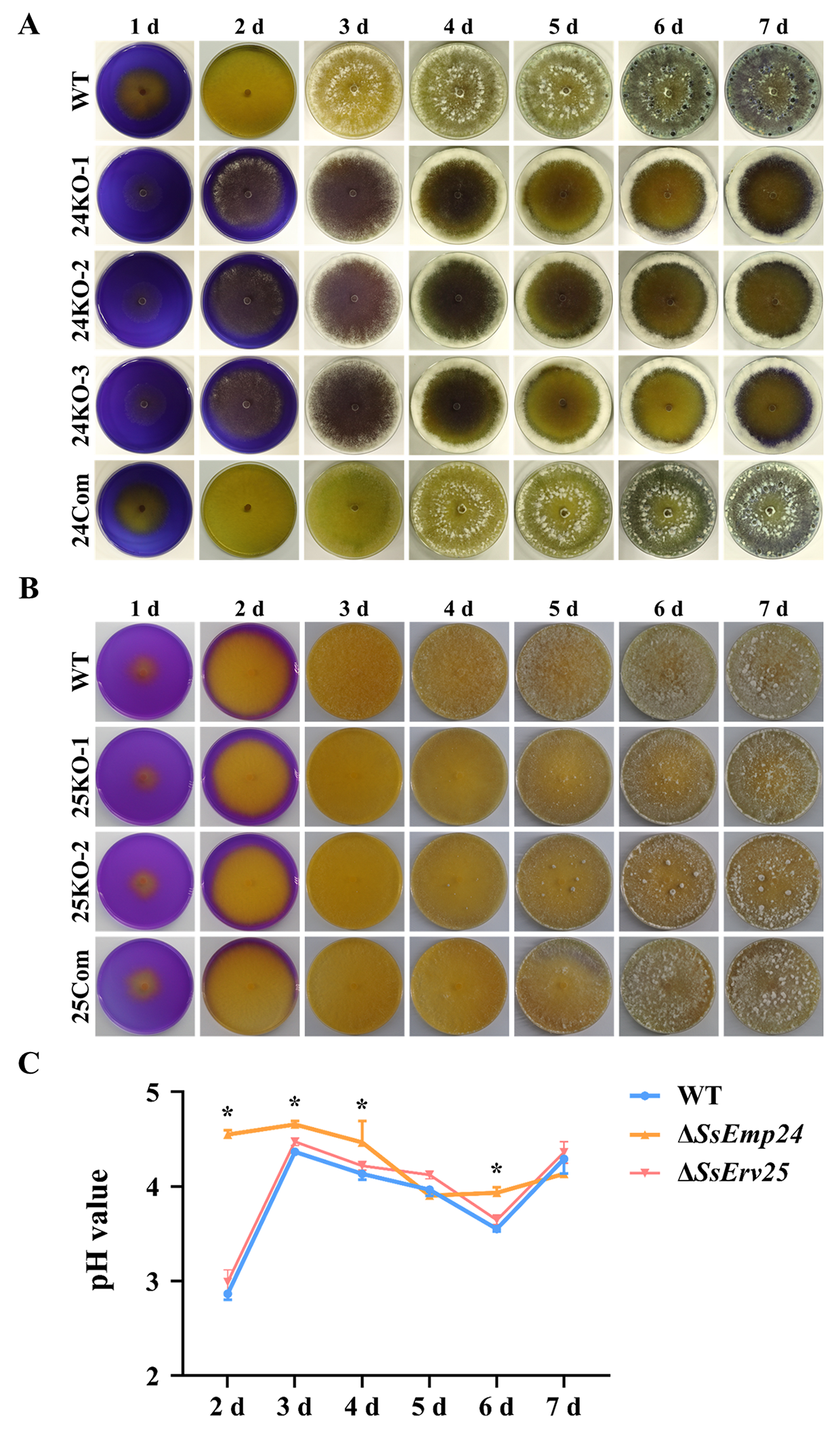

Supplement: FIG S4 [file mbio.03173-21-sf004.tif]

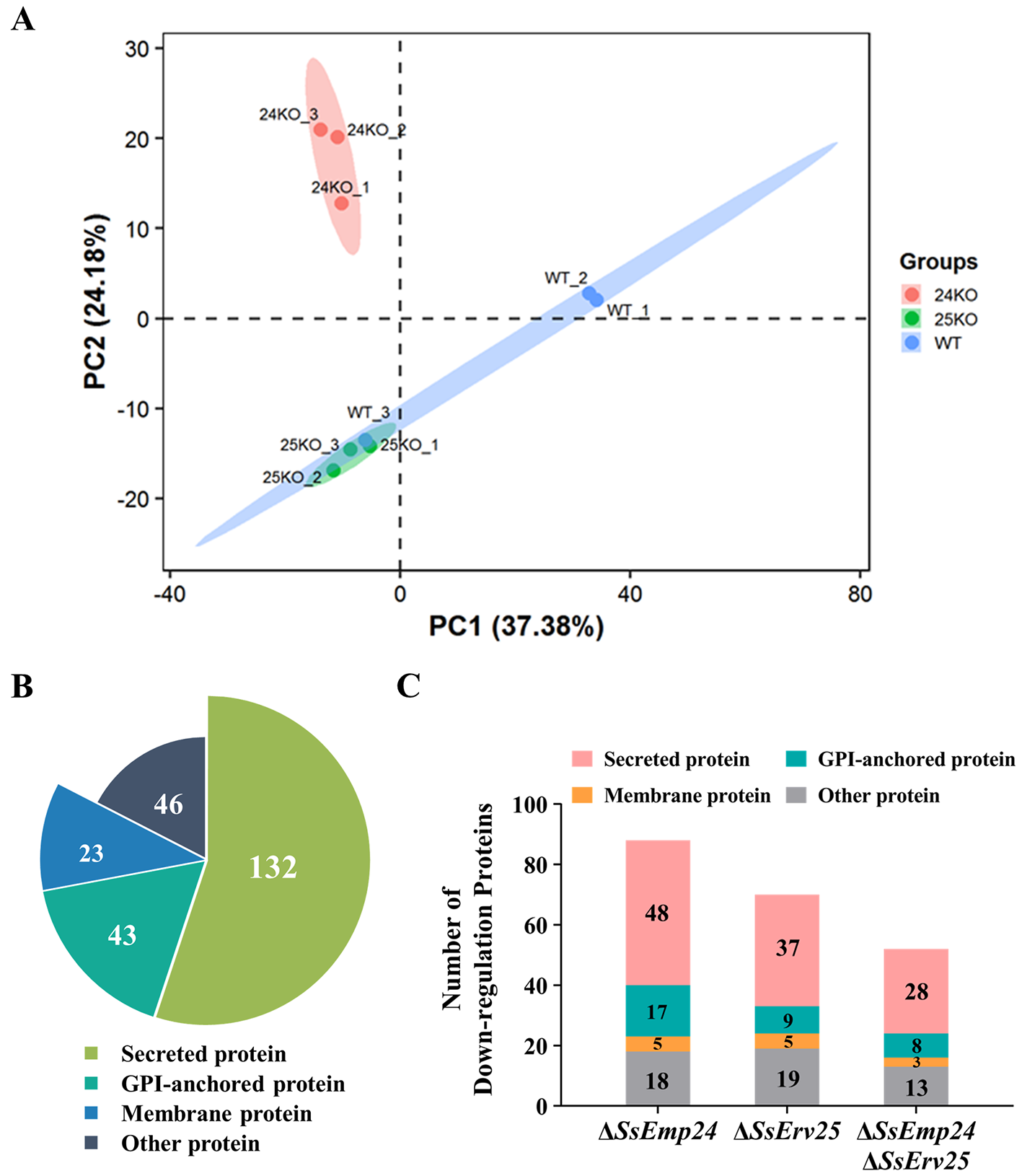

Supplement: FIG S5 [file mbio.03173-21-sf005.tif]

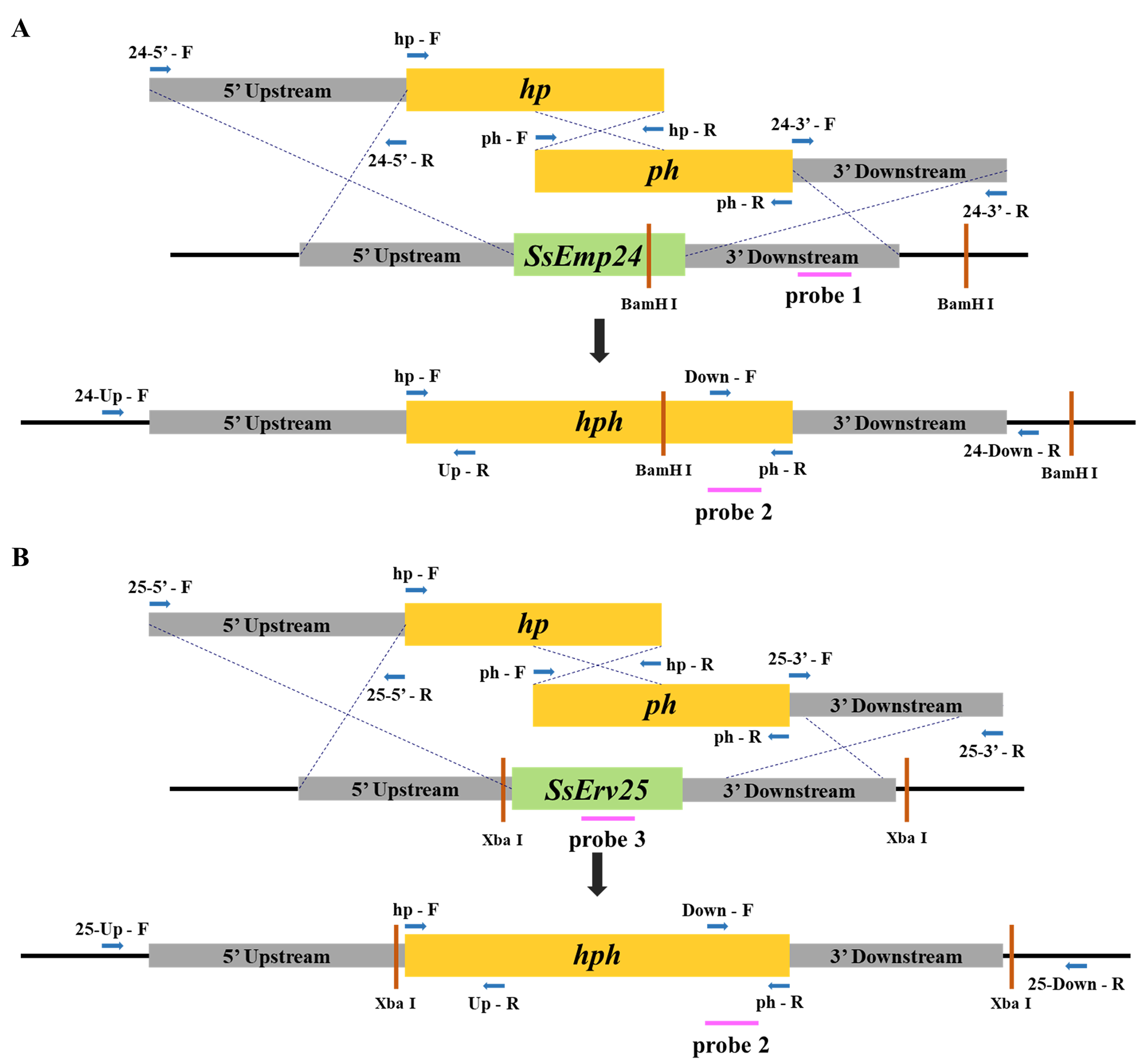

Supplement: FIG S6 [file mbio.03173-21-sf006.tif]
